# Peptide-functionalized nanoemulsions with exendin-4 as a model ligand

**DOI:** 10.1016/j.ijpx.2026.100598

**Published:** 2026-07-02

**Authors:** Natasa Holler, Bernd Gesslbauer, Carmen Tam-Amersdorfer, Ilse Letofsky-Papst, Claudia Mayrhofer, Fritz Andreae, Andreas Zimmer

**Affiliations:** a University of Graz, Institute of Pharmaceutical Sciences, Pharmaceutical Technology & Biopharmacy, Graz, Austria; b University of Graz, Institute of Pharmaceutical Sciences, Pharmaceutical Chemistry, Graz, Austria; c Medical University of Graz, Otto Loewi Research Centre – Institute of Immunology, Graz, Austria; d Institute of Electron Microscopy and Nanoanalysis, Graz University of Technology, NAWI Graz, Graz, Austria; e Center for Electron Microscopy, Graz, Austria; f piCHEM Forschungs- und Entwicklungs GmbH, Raaba-Grambach, Austria

**Keywords:** Drug targeting, Adipocytes, GLP-1, GLP-1 receptor, Nanoemulsion

## Abstract

Glucagon-like peptide-1 receptor (GLP-1R) agonists are increasingly explored for the treatment of obesity and metabolic disorders, yet the biological consequences of presenting these ligands on the surface of lipid-based nanocarriers remain insufficiently understood. In this study, exendin-4–functionalized nanoemulsions (ex-4 NE) were developed and evaluated in 3T3-L1 pre-adipocytes and adipocytes with respect to cellular uptake and metabolic activity. Both nanoemulsion types were efficiently internalized, however, ex-4 NE displayed higher uptake rates and larger AUC values than non-targeted PEG NE. Uptake was strongly influenced by differentiation stage but only minimally affected by GLP-1R inhibition. Chlorpromazine reduced uptake only in adipocytes differentiated in presence of exendin-4, indicating a shift toward clathrin-mediated routes upon chronic ex-4 treatment, but overall supporting that nanoemulsion uptake is not directly receptor-dependent. Colocalization analysis showed only modestly higher overlap of ex-4 NE with GLP-1R in comparison to the PEG NE.

Despite the absence of pronounced receptor-mediated internalization, ex-4 NE exerted substantially stronger GLP-1R–dependent metabolic effects than free exendin-4. When administered early in differentiation, ex-4 enhanced adipogenesis, whereas addition from day 2 markedly reduced lipid accumulation. Notably, 10 nM ex-4 NE produced effects comparable to 1000 nM free ex-4, suggesting enhanced receptor activation via high local ligand density at the nanoemulsion surface, enabling multivalent interactions. At higher NE concentrations, lipid accumulation increased due to lipid substrate supply, independent of GLP-1R signaling.

Taken together, these findings indicate that exendin-4 functionalization enhances nanoemulsion activity primarily through amplification of biological responses rather than through pronounced receptor-mediated uptake.

## Highlights

• Stable exendin-4-functionalized nanoemulsions were successfully developed.
• Exendin-4 surface functionalization increased nanoemulsion uptake in both 3T3-L1 pre-adipocytes and mature adipocytes.
• Chronic exendin-4 exposure during adipogenesis shifted nanoemulsion uptake partly toward clathrin-dependent endocytosis.
• Nanoemulsion-bound exendin-4 showed enhanced lipolytic effects in 3T3-L1 adipocytes compared to free peptide.

## Graphical abstract

**Figure:**
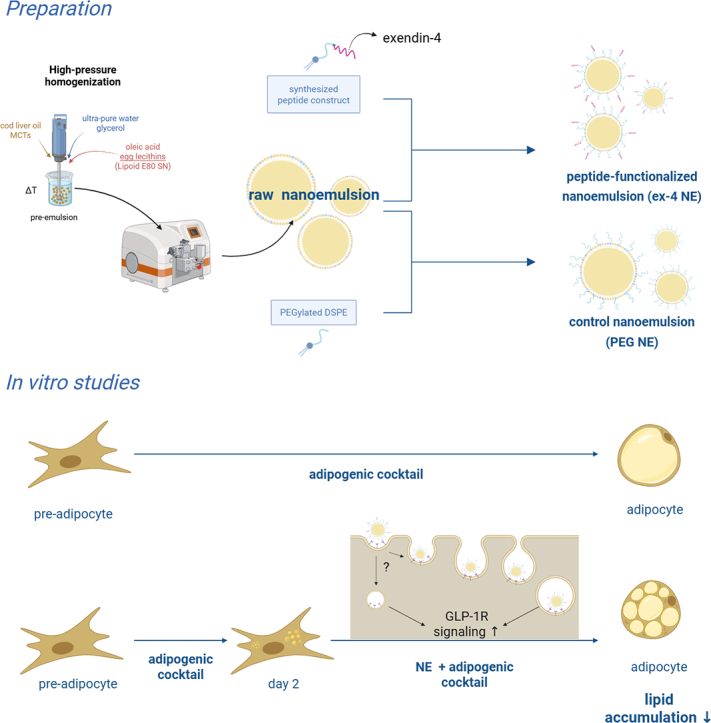
Created in BioRender. Holler, N. (2026) https://BioRender.com/cr2u2sg

## 1 Introduction

The prevalence of obesity in adults has more than doubled since 1990, whereas in 2022, 1 in 8 people in the world were obese. Responding to this escalating global crisis, the World Health Organization (WHO) issued its first global guideline in December 2025 on the safe, fair, and appropriate use of GLP-1 analogs for obesity treatment (WHO, 2025, WHO, 2025b).

Glucagon-like peptide-1 (GLP-1) is an incretin hormone that plays a central role in the postprandial insulin response. However, it is also important for the regulation of energy homeostasis, as it exerts pleiotropic effects across multiple organs and tissues. Its glucose-lowering actions are primarily mediated via GLP-1R in pancreatic β-cells. On the other hand, its weight-reducing effects are largely driven by centrally mediated appetite suppression and delayed gastric emptying, and are also mediated by GLP-1R in these tissues. Beyond glycemic control and weight reduction, emerging clinical and experimental evidence indicates further benefits, including improvements in cardiovascular function (e.g., cardiac output, heart rate, and blood pressure), enhanced insulin sensitivity in skeletal muscle and adipose tissue, and favorable effects on hepatic and renal function. Accordingly, GLP-1 analogues are increasingly recognized as integral therapeutic agents for obesity, with or without comorbidities such as cardiovascular disease, type 2 diabetes (T2DM), chronic kidney disease, hypertension, and metabolic dysfunction-associated steatotic hepatitis (MASH) (Bu et al., 2024; Holler et al., 2025; Müller et al., 2019).

Obesity is characterized by excessive fat accumulation and chronic low-grade inflammation of adipose tissue (AT), which is now recognized as a metabolically active endocrine organ with systemic effects (Cani and Van Hul, 2024). Accordingly, the regulation of adipose tissue metabolism is of central importance in the treatment of obesity. Emerging evidence indicates that GLP-1R expression in adipose tissue may be context-dependent, varying with insulin resistance, adipose depot, and treatment history. For example, higher GLP-1R expression has been reported in differentiated primary adipocytes from insulin-resistant (but not diabetic), morbidly obese individuals compared with lean donors, whereas studies on visceral adipose tissue (VAT) from patients with T2DM have described lower GLP-1R expression or reduced GLP-1 sensitivity (Ejarque et al., 2019; El Bekay et al., 2016). These discrepancies likely reflect cohort characteristics and methodological differences, which need to be explored further, as this may present an interesting approach for targeting adipose tissue in the obese individuals.

In this study, we present exendin-4-functionalized oil-in-water nanoemulsions (NEs) as a platform to investigate how surface presentation of a GLP-1R agonist influences cellular uptake and lipid metabolism in adipocytes. Nanoemulsions, due to their physical characteristics, present a great opportunity for delivery of hydrophobic drugs, different ways of administration (especially parenteral administration), and alternative uptake mechanisms comparing to the free drug (Tayeb and Sainsbury, 2018). Here, we employed the 3T3-L1 adipogenesis model as a proof-of-concept system to evaluate the uptake behavior of exendin-4-functionalized nanoemulsions and to assess their effects on intracellular lipid accumulation. Particular emphasis was placed on investigating the potential contribution of GLP-1R interactions to nanoemulsion uptake and biological activity, while recognizing that receptor expression and responsiveness may vary across differentiation stages and physiological conditions. Collectively, this work aims to provide insight into the interplay between ligand functionalization, cellular uptake pathways, and metabolic responses in adipocytes.

### 1.1 Effects of GLP-1 at different stages of adipogenesis

**Fig. 1:**
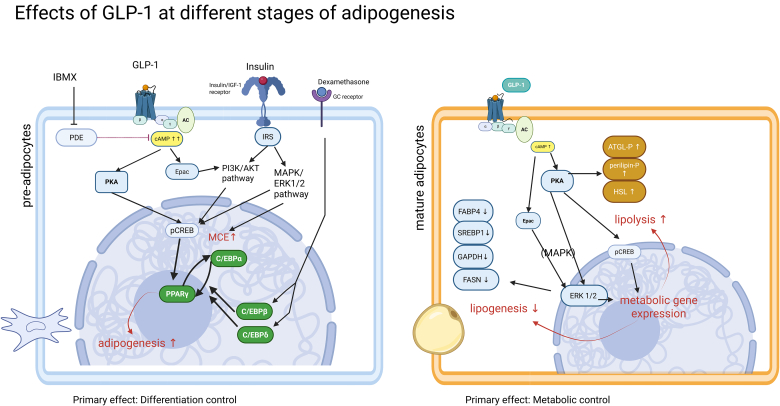
Effects of GLP-1 at different stages of adipogenesis. Abbreviations: IBMX (3-isobutyl-1-methylxanthine); PDE (phosphodiesterase); cAMP (cyclic adenosine monophosphate); Epac (exchange protein directly activated by cAMP); AC (adenylate cyclase); PKA (protein kinase A); PI3K (phosphoinositide 3-kinase); Akt (protein kinase B); MAPK (mitogen-activated protein kinase); Extracellular signal-regulated kinase (ERK); GC (glucocorticoid); pCREB (phosphorylated cAMP response element-binding protein); MCE (mitotic clonal expansion); PPARγ (peroxisome proliferator-activated receptor gamma); C/EBP (CCAAT/enhancer binding protein); FABP4 (fatty acid binding protein 4); SREBP1 (sterol regulatory element-binding protein-1); GAPDH (glyceraldehyde 3-phosphate dehydrogenase); FASN (fatty acid synthase); pATGL (phosphorylated adipose triglyceride lipase); perlipin-P (phosphorylated perlipin); HSL (hormone-sensitive lipase). Created in BioRender. Holler, N. (2026) https://BioRender.com/r0wvrzk

The 3T3-L1 cell line is a well-established model for studying adipogenesis and the intracellular pathways that regulate lipid metabolism. Adipogenesis is a highly coordinated, multi-stage program through which pre-adipocytes differentiate into mature lipid-rich adipocytes. The effects of GLP-1 on (pre-)adipocytes depend strongly on the stage of differentiation and the metabolic context and the main signaling cascades engaged by GLP-1 are schematically summarized in Fig. 1, where the effects in pre-adipocytes versus mature adipocytes are compared.

Differentiation of 3T3-L1 pre-adipocytes is typically induced with a cocktail containing insulin, 3-isobutyl-1-methylxanthine (IBMX), and dexamethasone. IBMX acts via cAMP signaling, leading to phosphorylation of cAMP-response element-binding protein (CREB) and induction of the key adipogenic transcription factors peroxisome proliferator-activated receptor gamma (PPARγ) and CCAAT/enhancer binding protein alpha (C/EBPα), which form a positive feedback loop that reinforces adipogenic differentiation. cAMP supports differentiation through protein kinase A (PKA) and the exchange protein directly activated by cAMP (Epac), with Epac being crucial for the full pro-adipogenic response to IBMX. Insulin promotes adipogenesis mainly via the insulin-like growth factor-1 (IGF-1) receptor, activating PI3K/Akt and MAPK/ERK pathways. Dexamethasone, acting through the glucocorticoid (GC) receptor, induces adipogenic transcription factors such as C/EBPβ and C/EBPδ and transiently limits pre-adipocyte proliferation. Although glucocorticoids can also enhance lipolysis by upregulating adipose triglyceride lipase (ATGL) and hormone-sensitive lipase (HSL), these longer-term catabolic effects are less relevant for short-term induction and are not depicted in Fig. 1 (Giroud et al., n.d.; Guo et al., 2024; Vali et al., 2024; Xu et al., 2009).

In the early phase of 3T3-L1 differentiation, GLP-1 has been reported to promote adipogenesis and to increase lipid storage (Chen et al., 2017). This pro-adipogenic action is mediated by GLP-1R and involves cAMP/PKA signaling, which increases CREB phosphorylation and enhances PPARγ expression.

In contrast, in mature 3T3-L1 adipocytes, GLP-1 generally reduces lipid accumulation by both inhibiting lipogenesis and promoting lipolysis. Liraglutide has been shown to decrease fatty acid synthase (FASN) expression in a dose-dependent manner, indicating suppression of de novo lipogenesis (Chen et al., 2017). Complementary findings demonstrate that GLP-1 signaling enhances lipolytic activity, as evidenced by increased ATGL phosphorylation and elevated glycerol/protein ratios in both 3T3-L1 and human adipocytes. Of note, these effects are attenuated by adenylate cyclase (AC) inhibition, implicating a cAMP-dependent mechanism (El Bekay et al., 2016; Vendrell et al., 2011). GLP-1 also increases the phosphorylation of perilipin, a lipid-droplet–coating protein that limits basal lipolysis while enabling cAMP/PKA-mediated lipolytic activation (Shen et al., 2009). In addition to these functional changes, a reduction in key lipogenic and lipid-regulatory proteins—including fatty acid binding protein 4 (FABP4), sterol regulatory element-binding protein-1 (SREBP1), and glyceraldehyde-3-phosphate dehydrogenase (GAPDH)—has also been reported, further supporting an overall anti-lipogenic profile for GLP-1 in mature adipocytes (Vendrell et al., 2011; Yang et al., 2013).

The impact of GLP-1 on insulin-related signaling appears to be highly context-dependent. Bekay et al. reported no increase in Akt or ERK1/2 phosphorylation in mature 3T3-L1 adipocytes following GLP-1R stimulation, whereas Yang et al. observed activation of Akt, p38, and ERK1/2 during early differentiation, although only pAkt levels remained elevated relative to non-treated controls (Yang et al., 2013). Mechanistically, some of these effects likely involve cAMP/Epac/PI3K signaling, while GLP-1–mediated reductions in FASN expression appear at least partially dependent on PKA and MAPK (ERK1/2) pathways (Chen et al., 2017).

Taken together, GLP-1 engages cAMP-dependent signaling to modulate MAPK/ERK and PI3K/Akt pathways in a stage-specific manner—supporting adipogenesis and lipid storage during early differentiation, while promoting lipolysis over lipogenesis in mature adipocytes. These coordinated effects may ultimately contribute to improved adipocyte insulin sensitivity, alongside the increased adiponectin expression and secretion commonly associated with GLP-1 action.

## 2 Materials and methods

### 2.1 Materials

Exendin-4-Cys-40-amide (exendin-4-Cys40-NH_2_) (piCHEM, Raaba-Grambach, Austria) and DSPE-PEG(2000)-maleimide (Avanti ® Polar Lipids, Alabaster, USA) were used as assemblies for the peptide construct. The emulsifiers used for the preparations of raw NEs were egg lecithins Lipoid ® E80 and E80 SN (Lipoid GmbH, Ludwigshafen, Germany), and soy lecithin PL90 (Fresenius Kabi, Brunna, Sweden). Other components used in the preparation of nanoemulsions were soybean oil, cod liver oil, olive oil, castor oil, Glycerol 85%, medium-chain triglycerides (MCTs) (Herba Chemosan, Graz, Austria), and oleic acid (VWR™, Avantor, USA).

For cell cultivation and differentiation, the following substances were used: low and high glucose Dulbecco's Modified Eagle Medium (lgDMEM and hgDMEM, Thermo Fischer Scientific); fetal bovine serum (FBS, Sigma Aldrich); 4-hydroxyethylpiperazine ethanesulfonic acid (HEPES, Thermo Fischer Scientific); l-glutamine (Thermo Fischer Scientific); penicillin-streptomycin (Thermo Fischer Scientific); Dexamethasone (Sigma Aldrich); 3-isobutyl-1-methylxanthine (IBMX, Sigma Aldrich); and insulin (Sigma Aldrich). Further information about purchased substances is given in the supplementary Materials.

### 2.2 Methods

#### 2.2.1 Preparation of raw nanoemulsions

**Fig. 2:**
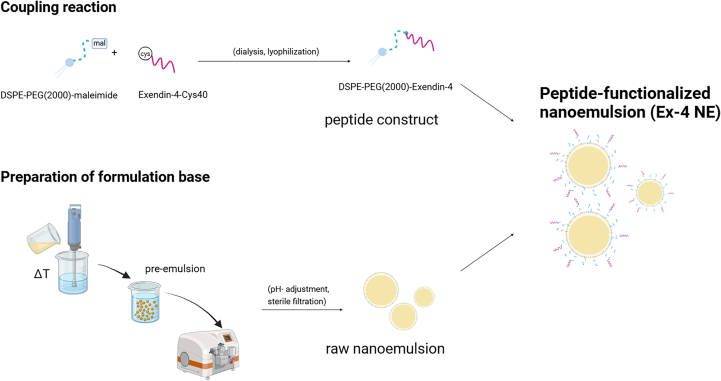
Schematical presentation of nanoemulsion preparation. Created in BioRender.com. Holler, N. (2026) https://BioRender.com/uz259sj

Nanoemulsions are established drug delivery systems for parenteral administration and are widely used in both parenteral nutrition and the delivery of hydrophobic drugs, such as propofol or diazepam (Diprivan®, Fresenius Kabi, Aspen Pharma; Diazemuls®, Actavis, Teva Pharmaceuticals) (Sobol et al., 2025). Previous work in our laboratory focused on the formulation of the raw nanoemulsion and examined factors influencing its stability and suitability for surface functionalization (results not published). These studies provided the foundation for the subsequent development of functionalized nanoemulsions. Because modifications at the oil-droplet surface can substantially affect dispersion stability, several formulation parameters were initially evaluated. In general, functionalized (ex-4) nanoemulsions were prepared by high-pressure homogenization (HPH) followed by conjugation with a previously synthesized peptide construct (see Fig. 2).

The prepared raw NEs differed in the type and concentration of emulsifier used, as well as in the composition of the oil phase. Variations in the oil phase specifically involved the choice of oil as a source of long-chain triglycerides (LCTs), and the mass ratio between long-chain and medium-chain triglycerides (LCT:MCT mass ratio). Each formulation was prepared using only one of the aforementioned emulsifiers, at either 1.4% or 1% (*w*/w). Detailed formulation composition is provided in the supplementary information. Initially, pre-emulsions were formed by mixing the pre-heated (70 °C) aqueous and oil components. The pre-emulsions underwent six cycles of high-pressure homogenization at 800 ± 20 bar, while maintaining the temperature at 70 °C (Panda 2K NS1001L Spezial, GEA Niro Soavi, Parma, Italy). The resulting nanoemulsions were then cooled to room temperature (∼23 °C), and the pH was adjusted to 8.5 using 0.1 N NaOH. Finally, the nanoemulsions were aliquoted, sterile-filtered (0.22 μm/25 mm cellulose acetate syringe filter, VWR®), and stored at 4 °C.

#### 2.2.2 Coupling reaction

Exendin-4-Cys40-NH_2_, which contains an additional cysteine group at the N-terminus, was coupled to DSPE-PEG(2000)-maleimide via a maleimide-thiol reaction. First, the peptide was incubated at 56 °C with a 20-fold molar excess of the reducing agent, 10 mM TCEP, for 20 min to reduce disulfide bonds. It was then transferred to 12 mL of reaction buffer (10 mM HEPES, degassed and adjusted to pH 7.0) at room temperature. Subsequently, a 5-fold molar excess of DSPE-PEG(2000)-maleimide (0.05 mg/mL in DMSO) was added dropwise to the reaction buffer under constant stirring in a nitrogen atmosphere. The reaction proceeded for 2 h. To quench excess maleimide groups, a freshly prepared solution of L-cysteine (0.1 mg/μL, 50-fold molar excess) was added and the mixture was stirred for an additional 30 min. Upon completion, excess reagents were removed by dialysis using a 2000 MWCO Slide-A-Lyzer® Dialysis cassette (Pierce Biotechnology, Thermo Scientific). Dialysis was performed with a 200-fold volume of dialysis buffer (10 mM HEPES, pH 7.4) over three cycles at 4 °C, with each cycle lasting 2 h. In the final step, the product solution was aliquoted into 2 mL-vials, 4% (*w*/w) mannitol was added as a cryoprotectant and the solution was lyophilized for 48–72 h.

#### 2.2.3 Preparation of the peptide-directed NE and pegylated (Control) NE

For the peptide conjugation, the lyophilized DSPE-PEG(2000)-exendin-4 product was mixed with the selected raw nanoemulsion so that the molar ratio between unmodified lecithin (egg lecithin E80 SN) and the DSPE-PEG(2000)-exendin-4 was 1:75. Accordingly, the overall molar ratio of total pegylated phospholipids to unpegylated phospholipids was set at 1:15. The control nanoemulsion, containing only PEG chains on the oil droplet surfaces (without peptide), was prepared by mixing DSPE-PEG(2000) with the same batch of raw nanoemulsion, maintaining the same molar ratio between pegylated and unpegylated phospholipids as in the peptide-targeted formulation.

The selected PEG and ligand densities were based on literature data as well as our preliminary (unpublished) studies on comparable nanoemulsion formulations (Hak et al., 2012). Hak et al. reported that PEG surface density strongly influences ligand accessibility and targeting efficiency, with PEG concentrations above approximately 10 mol% promoting a brush-like conformation that may sterically hinder receptor interactions. Based on these findings, a DSPE-PEG(2000) content of approximately 6.25 mol% (1:15 ratio relative to unmodified phospholipids) was selected. The concentration of DSPE-PEG(2000)-exendin-4 corresponded to approximately 1.25 mol% of the total phospholipid content (1:75 ratio relative to unmodified phospholipids). Although Hak et al. employed a higher ligand density (2.5 mol%), their model ligand (RGD peptide) was substantially smaller than exendin-4. Therefore, a lower ligand density was chosen to minimize potential steric crowding while maintaining sufficient surface presentation of the peptide.

#### 2.2.4 Particle size distribution and Zeta potential

To assess the physical stability of the nanoemulsions (NEs), particle size distribution and zeta potential were measured using a Zetasizer Nano ZS (Malvern Panalytical, UK). The raw nanoemulsions were analyzed immediately after preparation, and subsequently after one week and three weeks (*n* = 3). Following pegylation, measurements were taken again immediately and after two weeks. After peptide conjugation, particle size distribution and zeta potential were assessed immediately, after one week, and after three weeks. For long-term stability assessment, selected batches used for in vitro studies were also analyzed up to two months after conjugation.

#### 2.2.5 Coupling reaction efficiency using liquid chromatography−mass spectrometry

The efficiency and quality of the coupling reaction between the peptide and the pegylated phospholipid was analyzed using nano liquid chromatography-mass spectrometry (nanoLC-MS), examining both the free peptide and the peptide conjugate.

Analyses were performed on an UltiMate 3000 RSLCnano system coupled to an LTQ-XL linear ion trap mass spectrometer (Thermo Fisher Scientific, Waltham, MA, USA) as previously described (Gesslbauer et al., 2018) The mobile phases for separation were: (A) 99.9% (*v*/v) water (HPLC grade, VWR) and 0.1% (v/v) formic acid; and (B) 80% (v/v) acetonitrile (HPLC grade, VWR) and 0.08% (v/v) formic acid. The mobile phase for the loading pump was water with 0.05% (v/v) trifluoroacetic acid (TFA). Samples were diluted in 0.05% TFA to 2.5 ng/μL, and 80 μL (∼200 ng peptide) were injected on a 300 μm id × 5 mm PepMap C18 trap column (Thermo Fisher Scientific) at a flow rate of 20 μL/min. After sample loading and desalting for 10 min, analytes were eluted from the trap column and separated on an Acclaim PepMap RSLC column (C18, 75 μm × 150 mm, 2 μm, 100 Å; Thermo Fisher Scientific) using a linear gradient from 25% to 90% solvent B over 70 min, followed by a 25-min wash at 90% B, and re-equilibration at 25% B for 15 min at a flow rate of 200 nL/min. The column oven was operated at 30 °C.

Eluted peptides were ionized via stainless steel emitters using a Nanospray Flex™ ion source (Thermo Fisher Scientific) and directly introduced into the LTQ XL mass spectrometer. The following electrospray ionization parameters were used: spray voltage, 1.7 kV; capillary temperature, 200 °C; capillary voltage, 30 V. Spectra were recorded in profile mode using Xcalibur software. Raw data (UV and MS) were processed using Freestyle software, version 1.7.73.12 (Thermo Scientific).

#### 2.2.6 Cryo-TEM

Cryogenic transmission electron microscopy (cryo-TEM) was used to analyze the morphology of oil droplets within both raw and peptide-conjugated nanoemulsions, using an FEI Tecnai T-12 microscope (FEI Company, Hillsboro, OR, USA). Samples were examined either undiluted or at a 1:1 dilution. Prior to application, holey carbon-coated copper grids (3.05 mm, 400 mesh) were glow-discharged for 5 s in an SC7620 mini sputter coater (Quorum Technologies Ltd., Laughton, UK). The grids were subsequently transferred into a Leica® EM GP plunge freezer (Leica Microsystems, Wetzlar, Germany), with the chamber set to 100% humidity and 22 °C. A 2.5 μL aliquot of the sample was applied onto the grid. After removing excess sample with blotting paper, the grid was swiftly plunged into liquid ethane (∼ − 182 °C) for vitrification. The vitrified grid was transferred to a liquid nitrogen reservoir and kept there until insertion into the cryo-holder. Once prepared, the cryo-holder with the grid was placed in the microscope and images were captured using a Gatan BioScan CCD camera (Model 792), whereas data were processed using Digital Micrograph software (Gatan, Pleasanton, CA, USA).

#### 2.2.7 In vitro studies

3T3-L1 pre-adipocytes, an established cell line for studying adipocyte differentiation, were used for in vitro experiments. Cells were cultivated in proliferation medium composed of low-glucose DMEM (lgDMEM), 10% fetal bovine serum (FBS), 1% l-glutamine, 1% HEPES, and 1% penicillin-streptomycin. Cells were seeded onto either 24-well or 96-well plates, and upon reaching confluence, differentiation was induced the following day using high-glucose DMEM (hgDMEM) with 10% FBS, 1% l-glutamine, 1% HEPES, 1% penicillin-streptomycin, IBMX (final concentration 500 μM), dexamethasone (final concentration 1 μM), and insulin (final concentration 10 μg/mL). On the second day, differentiation medium was introduced to each well, doubling the total medium volume. Differentiation medium excluded IBMX and dexamethasone and contained reduced insulin (5 μg/mL). To maintain differentiation, 50% of culture medium was replaced every other day with freshly prepared differentiation medium, maintaining consistent volume throughout the experiment. Cells were differentiated for 6–10 days, depending on the experiment.

#### 2.2.8 Cytotoxicity (MTS and LDH assays)

Cytotoxicity of the nanoemulsions and the uptake inhibitors was assessed using lactate dehydrogenase (LDH) leakage (CytoTox-ONE™ Homogeneous Membrane Integrity Assay, Promega®) and cell viability was determined by MTS assay (CellTiter 96® AQueous One Solution Proliferation Assay, Promega®). 3T3-L1 cells were seeded onto 96-well plates (Greiner Bio-One) at 7000 cells/well. The following day, cells were incubated for 4 h with nanoemulsion formulations or other test substances diluted in lgDMEM (without phenol red) across a specified concentration range. Assays were performed according to manufacturer's protocol. Briefly, for the LDH assay, 75 μL of supernatant was transferred to white 96-well plates, followed by addition of 75 μL CytoTox reagent and incubation for 30 min at 37 °C. Then, 37.5 μL stop solution was added, and fluorescence was measured at λEx/λEm 560/590 nm. For the MTS assay, cells were washed with PBS, then 100 μL/well lgDMEM (without phenol red) and 20 μL MTS reagent were added. Cells were incubated for 4 h, and absorbance was measured at 490 nm.

Cytotoxicity and cell viability were calculated according to Eqs. 1 and 2, respectively. Measurements were performed in triplicate. For positive control, 2 μL of 9% Triton X-100 in PBS was added per well. Negative control consisted of cells incubated with lgDMEM (without phenol red) alone.

(1) $$\mathrm{Percent Cytotoxicity}=100\times \left(\mathrm{Treated cells}-\mathrm{Negative Control}\right)/\left(\mathrm{Positive Control}-\mathrm{Negative Control}\right)$$

(2) $$\mathrm{Percent Cell Viability}=100\times A_{treated\;cells}/A_{negative\;control}$$

#### 2.2.9 Uptake studies

For uptake studies, 7 × 10^3^ cells per well were seeded into 96-well plates. The next day, cells were induced according to the differentiation protocol, while parallel wells of undifferentiated cells were maintained in supplemented lgDMEM, refreshed every two days. On day 6, cells were incubated with nanoemulsions. Pre-stained nanoemulsions (Nile Red, 10 μg/mL) were diluted in unsupplemented lgDMEM (without phenol red) to a final exendin-4 concentration of either 500 nM or 1000 nM, and incubated for times ranging from 15 min to 24 h. Control PEG nanoemulsions were diluted identically, maintaining equal concentrations for both groups. After incubation, nanoemulsions were removed, cells were washed with pre-warmed PBS, and lgDMEM (without phenol red) was added. Fluorescence was measured using a plate reader (CLARIOstar® plus, BMG LABTECH, Germany) at λEx/λEm 528–58/616–48 in matrix scan mode (5 × 5). Uptake was calculated relative to the standard (500 or 1000 nM exendin-4 defined as 100%). Measurements were conducted in triplicate. After live cell measurements, cells were treated with 0.1% Triton X-100 in 0.2 N NaOH/PBS to remove oil droplets bound to the cell surface and measure residual (internalized) nanoemulsion. Early uptake kinetics were analyzed by fitting to a one-phase association (pseudo-first-order) model (see Eqs. 3–6);

(3) $$Y\left(t\right)=Y_{0}+\left(\mathrm{Plateau}-Y_{0}\;\right)∙\left(1-e^{-Kt}\right)$$

(4) $$\mathrm{Half}-\mathrm{time}=\left(\ln\left(2\right)\right)/K$$

(5) $$\mathrm{Span}=\mathrm{Plateau}-Y_{0}$$

(6) $$\mathrm{Initial}\;\mathrm{rate}=K\times \left(\mathrm{Plateau}-Y_{0}\right)$$

where Y(t) is the uptake at time t, Y₀ is the uptake at the initial time point 0, Plateau (Y_max_) represents the maximal uptake achieved, and K is the uptake rate constant.

#### 2.2.10 Cell-uptake imaging, receptor imaging, and colocalization studies

For confocal microscopic imaging (Leica Microsystems), 70,000 3T3-L1 cells were seeded in 35 mm-diameter glass-bottom dishes (WillCo Wells B.V.) one day prior to differentiation. Cells were differentiated for 6 days as described above. Nanoemulsions were pre-stained with 10 μg Nile Red per mL prior to uptake experiments, initiated using double concentration for 15 min, then diluted to 1 μM exendin-4 with lgDMEM (without phenol red). After incubation, cells were washed, fixed in 4% formaldehyde for 10 min, permeabilized with 0.1% Triton X-100 in PBS for 3 min, and stained with AF488-phalloidin/Hoechst in 1% BSA/PBS for 20 min. Non-incubated cells stained identically for nuclei and cytoskeleton served as a negative control. After each step, cells were washed three times with PBS and finally mounted for imaging. Cells were visualized by confocal laser scanning microscopy (CLSM), and z-stack images were obtained.

For GLP-1R imaging, 3T3-L1 cells were seeded in chamber-divided culture slides (8-chamber flexiPerm®, Sarstedt) at 4000 cells/chamber, and differentiated for 6 days. Half of seeded slides with undifferentiated cells were frozen at −20 °C until day 6, then thawed and both groups stained identically. Cells were washed, air-dried, and fixed in 4% PFA/PBS for 20 min. After three wash steps with 0.05% Tween-20/PBS and treatment with 0.1% Triton X-100/PBS, cells were blocked in 5% donkey serum/5% BSA in PBS for 1 h. Primary antibody (αRbGLP1-R, 1 mg/mL, NovusBio) in 1:60 dilution was applied overnight at 4 °C. An isotype control (normal rabbit IgG, Calbiochem) was diluted to the same concentration and applied under identical conditions. The next day, after PBS washing, secondary antibody (DαRb AF 594) in 1:300 dilution was applied for 30 min. Nuclear staining with DAPI (1:1000) for 15 min was followed by mounting. Imaging was performed by CLSM with z-stack acquisition. All images were acquired using identical microscope settings (laser power, detector gain, pinhole, and scan parameters) for control and treated samples.

For colocalization studies, cells were seeded as above. The day after, cells were incubated with nanoemulsions pre-stained with 10 mM BODIPY™ 493/503 lipid stain (prepared as 100× stock), diluted to 1 μM exendin-4. After incubation, cells were washed, fixed with 4% PFA/PBS, and GLP-1R was immunostained as previously described. Imaging was performed by CLSM with z-stack acquisition and analysis done in ImageJ (JACoP plugin v2.1.4).

The Pearson correlation coefficient (PCC) was calculated according to Eq. 7, where n represents the number of pixel pairs analyzed, and x and y correspond to the fluorescence intensities of the compared channels (representing NE and receptor). The PCC measures the linear correlation between the fluorescence intensities of two channels across all pixels within the analyzed region. Values range from −1 to +1, where positive values indicate spatial correlation between the two signals.

(7) $$r=\frac{n\sum \mathrm{xy}-(\sum x)(\sum y)}{\sqrt{\left[n\sum x^{2}-{\left(\sum x\right)}^{2}\right]\left[n\sum y^{2}-{\left(\sum y\right)}^{2}\right]}}$$

Manders' coefficients M1 and M2 were also calculated to quantify fluorescence overlap; unlike PCC, Manders' coefficients are less sensitive to intensity differences and measure the fraction of one signal overlapping the other (Eqs. 8 and 9).

(8) $$M_{1}=\frac{\sum \mathrm{Intensities of Channel}\;A\;\mathrm{colocalized with Channel}\;B}{\sum \mathrm{Intensities of Channel}\;A}$$

(9) $$M_{2}=\frac{\sum \mathrm{Intensities of Channel}\;B\;\mathrm{colocalized with Channel}\;A}{\sum \mathrm{Intensities of Channel}\;B}$$

All images, including controls, were acquired using identical CLSM settings within each experiment.

#### 2.2.11 Uptake Inhibitors

To assess the effect of receptor inhibition on targeted nanoemulsion uptake, in vitro studies were conducted in 3T3-L1 cells using the GLP-1R antagonist exendin(9–39) (ThermoFischer Scientific, J66126.MCR) and chlorpromazine (Sigma Aldrich, C8138), an inhibitor of clathrin-mediated endocytosis. Experiments were performed in differentiated 3T3-L1 adipocytes after 6 days of differentiation. To model a state of persistent GLP-1R signaling during adipogenesis, cells were chronically exposed to 100 nM exendin-4 throughout the differentiation period. This approach is known to alter lipid metabolism and endocytic pathway usage (Chen et al., 2017) Cells were seeded into 24-well plates (5000 cells/well) three days before induction of differentiation. After reaching confluence, differentiation was induced as described above. Nanoemulsions were pre-stained with BODIPY 493/503 lipid stain, diluted in low-glucose DMEM to a final concentration corresponding to 500 nM exendin-4; and control PEG nanoemulsions were diluted identically. On day 6, cells were washed with PBS, pre-incubated with inhibitor solutions prepared in low-glucose DMEM for 1 h, then incubated with nanoemulsions and inhibitors together for an additional hour. After incubation, cells were washed, fixed with 4% formaldehyde for 10 min, permeabilized with 0.1% Triton X-100/PBS for 3 min, and nuclei were stained with DAPI (1:1000) for 20 min. Cells were washed with PBS after every step and mounting medium was added at the end. Fluorescence was measured in matrix scan mode (15 × 15) at excitation/emission wavelengths of 360/460 nm for DAPI and 490/530 nm for nanoemulsions (BODIPY 493/503).

#### 2.2.12 Oil red O (ORO) staining

Lipid accumulation was assessed by Oil Red O staining after 10 days of differentiation. To determine the effect of GLP-1 analog on lipid accumulation, exendin-4 was added to the culture medium at concentrations ranging from 10 to 1000 nM. On day 10, cells were washed with PBS, fixed using 4% formaldehyde first for 10 min and then again for 1 h, washed, incubated with 60% isopropanol for 5 min, air-dried, and stained with ORO working solution for 10 min. After three washes with MQ-water, cells were photographed under a Zeiss Axio Observer Z1 with Axiocam ERc5s at 200× and 400× magnification. Afterwards, cells were washed once with 60% isopropanol and dye was eluted for 5 min with 100% isopropanol. The eluent was transferred to a new plate and absorbance at 492 nm was measured. Undifferentiated cells served as control. Measurements were performed in triplicate; droplet size analysis involved at least four images (400× magnification) per sample, i.e., 12–14 images per group. Image analysis was performed in ImageJ v1.54p as previously described (Kaczmarek et al., 2024).

## 3 Results

### 3.1 Size and zeta potential of raw nanoemulsions during preformulation

The prepared raw nanoemulsions (NEs) differed with respect to the type and concentration of emulsifier used (1.0% or 1.4%), the type of oil serving as a source of long-chain triglycerides (LCTs), and the mass ratio of medium-chain to long-chain triglycerides (MCT:LCT). Variations in lecithin mixtures were based primarily on source, i.e., egg (E80 and E80SN) or soybean (PL90), and the ratio of phosphatidylcholine (PC) to other phospholipids (primarily phosphatidylethanolamine). According to manufacturer's certificates, the PC content accounts for 80%, 70%, and 60–80% of total phospholipids in E80, E80SN, and PL90, respectively.

**Fig. 3:**
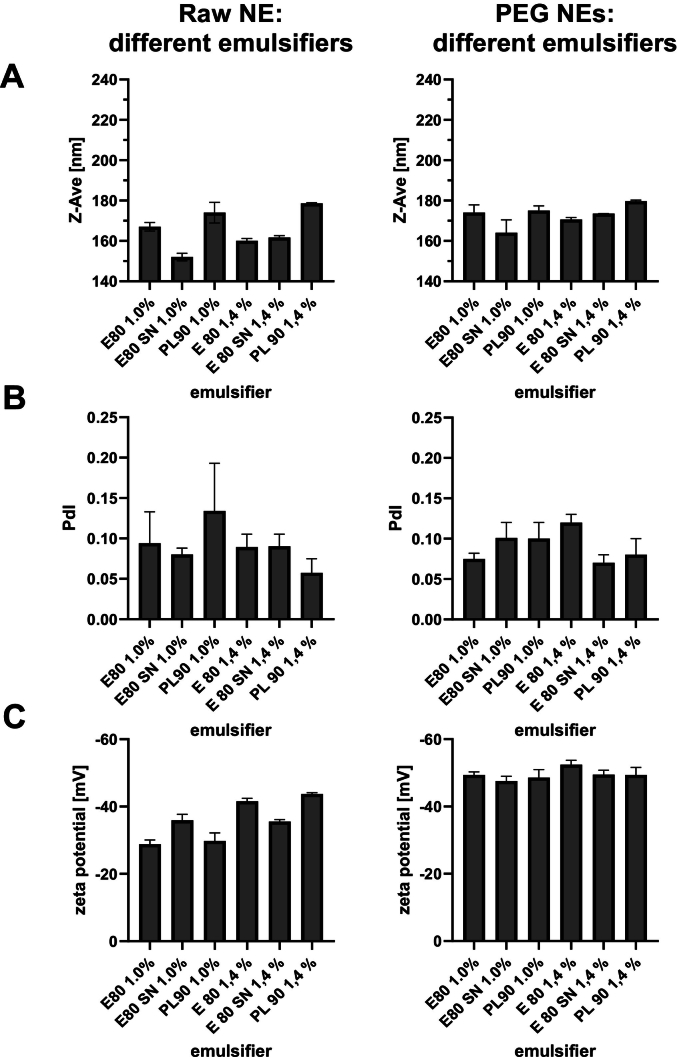
Effect of different emulsifiers on oil droplet size (A), size distribution (B) and zeta potential (C). All NEs were prepared using the same oil phase (soybean oil as the LCTs source, MCT:LCT mass ratio 1:1). Results are presented as mean ± SD (*n* = 3).

The first objective was to determine whether the emulsifier concentration could be reduced to 1% without compromising the stability of the raw or PEG NEs. We also aimed to evaluate whether different emulsifier mixtures affected droplet size, size distribution, or zeta potential (Fig. 3). For each raw NE, a corresponding PEGylated form was also prepared to compare overall stability. The NE with E80SN exhibited the smallest mean droplet size, and therefore this emulsifier was selected for further development.

**Fig. 4:**
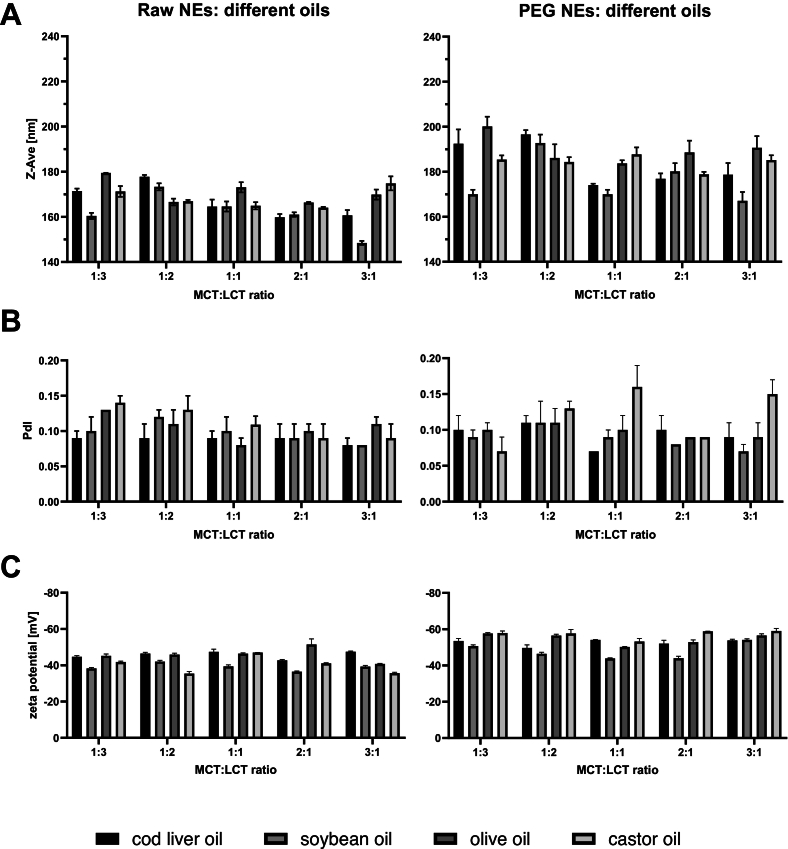
Effect of different oil phases on size (A), size distribution (B), and zeta potential (C) of oil droplets. All NEs were prepared with 1% E80SN as emulsifier, differing only in the oil phase (type of LCT source and MCT:LCT mass ratios). Results are presented as mean ± SD (*n* = 3).

Next, we assessed how changes in the oil phase, more specifically the type of oil as an LCT source and the MCT:LCT ratio, affected the aforementioned physical parameters (Fig. 4). All tested formulations exhibited particle sizes in the nanometer range with relatively narrow size distributions and stable zeta potentials. Particle size is a critical parameter for parenteral nanoemulsions and is generally expected to fall within a defined range (usually 100–500 nm), where both excessively large droplets and very small particles may be undesirable (Sadu Singh et al., 2020; Trinick and Duly, 2012). Cod liver oil was selected as the oil phase for subsequent formulations due to its consistent and homogeneous particle size distribution (PdI). An MCT:LCT ratio of 1:1 resulted in the smallest particle size and was therefore chosen for further studies. Across all tested formulations, the *Z*-average size ranged from 150 to 200 nm, with PdI values between 0.10 and 0.20. The zeta potential of PEGylated formulations was approximately −50 mV, indicating good colloidal stability. These physicochemical characteristics fall within ranges generally considered acceptable for nanoemulsion-based systems intended for parenteral administration.

Pharmacopoeial guidelines for lipid injectable emulsions primarily focus on the control of large-diameter droplets rather than mean particle size. The European Pharmacopoeia (Ph. Eur. 2.9.19) and the United States Pharmacopeia provide methods to assess particulate matter and globule size distribution, emphasizing the limitation of large particles. In particular, USP <729> specifies that the mean droplet diameter (MDD) should be below 500 nm and that the volume-weighted fraction of fat globules larger than 5 μm (PFAT5) should not exceed 0.05%, typically determined by light obscuration techniques. In this study, pharmacopoeial compliance was not evaluated in a strict sense, as the primary focus was on targeted nanocarrier development rather than formulation of clinically approved parenteral emulsions. Instead, these guidelines were used as a general reference framework. Dynamic light scattering (DLS) was applied as a screening method to assess particle size and distribution. While DLS is well suited for nanometer-scale measurements, it is inherently less sensitive to detecting low-abundance micrometer-sized particles and therefore cannot reliably assess large-droplet fractions such as PFAT5.

### 3.2 Size and zeta potential after PEGylation and peptide conjugation

**Fig. 5:**
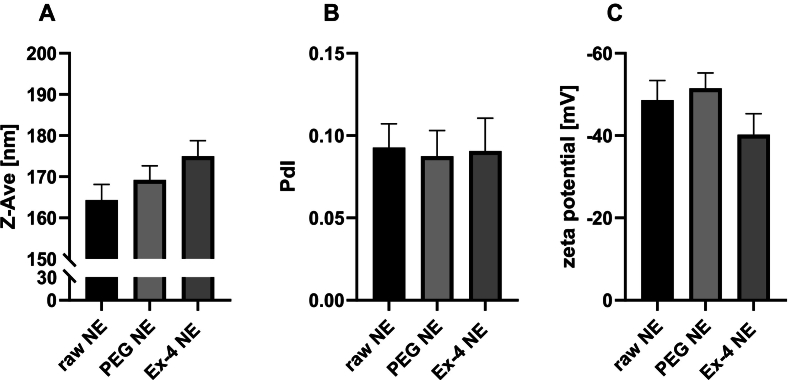
Particle size (A), size distribution (B), and zeta potential (C) of NEs before and after conjugation. Values represent mean ± SD, *n* = 9 across three different NE batch series.

Conjugated NEs were analyzed for particle size and zeta potential (Fig. 5). As expected, the size of the oil droplets increased slightly (∼5–10 nm) after conjugation. The conjugation did not noticeably affect the size distribution (Fig. 5B), but did lead to a slight shift in zeta potential (Fig. 5C). For ex-4 NE, a minor decrease in zeta potential was observed, likely due to surface modification by the conjugated peptide and residual reaction buffer (10 mM HEPES) in the lyophilized product. The presence of HEPES may increase the ionic strength of the surrounding medium, which can compress the electrical double layer and thereby influence the measured zeta potential.

### 3.3 Stability of conjugated nanoemulsions

**Fig. 6:**
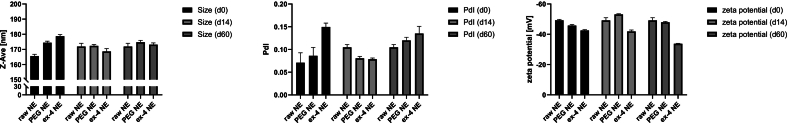
Stability of conjugated NEs compared to raw NEs after two weeks and after two months. Data represent mean ± SD (*n* = 3).

Dynamic light scattering (DLS) measurements were also conducted to examine the stability of the NEs over time. During preformulation, raw NEs were analyzed one and three weeks after preparation, while PEGylated NEs were tested after two weeks (results of individual batches are provided in Supplementary Materials). Selected batches of conjugated NEs (ex-4 NE and PEG NE) were monitored for up to two months and an example is shown in Fig. 6. Ex-4 NE exhibited lower long-term stability compared to raw and PEG NEs, as indicated by a notable decrease in zeta potential after two months. Nonetheless, all NEs remained stable for at least several weeks under storage. *For* in vitro studies, fresh batches were always prepared and used within two weeks.

### 3.4 Coupling reaction

**Fig. 7:**
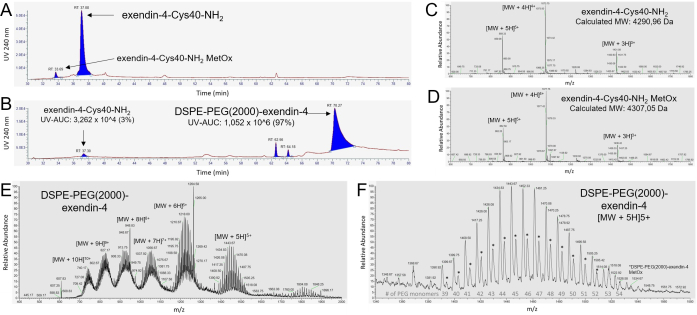
Exendin-4-Cys40-NH_2_ is effectively coupled to DSPE-PEG(2000). UV chromatogram (214 nm) of exendin-4-Cys40-NH_2_ prior to reaction (A). UV chromatogram (214 nm) of the reaction product, DSPE-PEG(2000)-exendin-4, showing ∼97% of the peptide was successfully conjugated to the pegylated phospholipid (B). ESI mass spectra of exendin-4-Cys40-NH_2_ before reaction (C, D). ESI mass spectra of DSPE-PEG(2000)-exendin-4 after reaction, demonstrating the presence of conjugates with 39–54 PEG monomers (E, F). Minor amounts of extendin-4 contain an oxidized methionine (A, D and F). UV chromatographic peak areas (UV-AUCs) were calculated with Freestyle software using the Genesis peak integration algorithm.

The coupling reaction between exendin-4 and DSPE-PEG(2000) was highly efficient, resulting in approximately 97% of the peptide conjugated to the phospholipid (Fig. 7).

### 3.5 Cryo-TEM

**Fig. 8:**
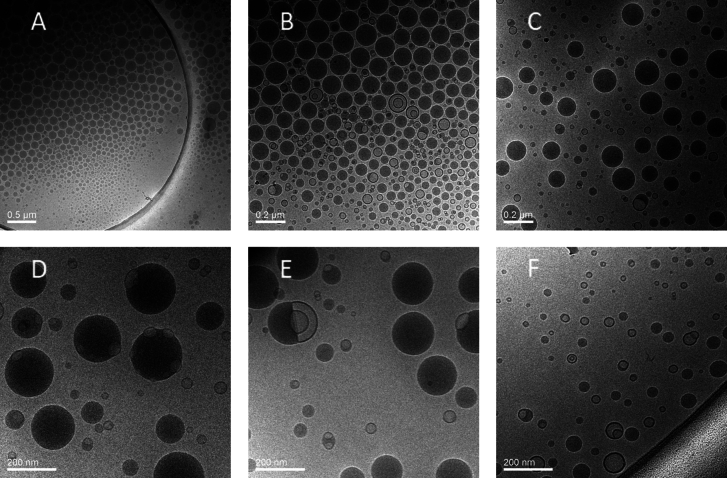
Cryo-TEM images of the raw nanoemulsion. Dark spots correspond to oil droplets, while transparent vesicular forms indicate liposomes or micelles. Aggregation between droplets and vesicles can occur (see panels D–E). Scale bars: 0.5–0.2 μm.

**Fig. 9:**
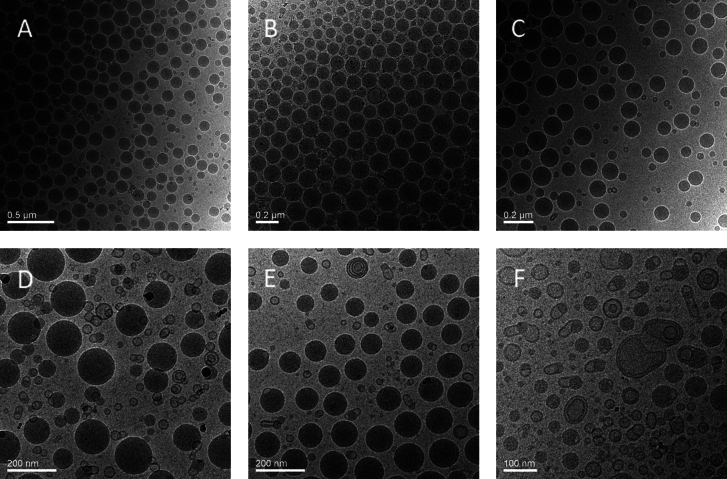
Cryo-TEM images of the exendin-4 nanoemulsion. Dark spots correspond to oil droplets, while transparent vesicular forms indicate liposomes or micelles. Aggregation between droplets and vesicles can occur (see panels E–F). Scale bars: 0.5–0.1 μm.

Cryo-TEM images were acquired for both the raw NE and ex-4 NE from the same batch. No apparent differences were observed between the raw NE (Fig. 8) and the ex-4 NE (Fig. 9). Besides oil droplet, vesicular structures were also present; depending on their size, these represent either liposomes (∼ 100–150 nm), or micelles (∼ 40 − 80 nm). Aggregates of oil droplets and vesicles were also sometimes observed.

As the emulsifier concentration was intentionally reduced in the raw nanoemulsions (NEs) to minimize micelle formation and favor the formation of NE droplets, the increase in emulsifier concentration during conjugation may shift this balance toward increased micelle formation, which is unfavorable in terms of precise dosing of hydrophobic drugs. Cryo-TEM analysis revealed the presence of a minor population of vesicular structures in both cases, before and after conjugation. However, DLS measurements showed an increase in hydrodynamic diameter after conjugation. This suggests that micelle formation was not the dominant structural change in the system. Instead, the increase in hydrodynamic diameter is more likely attributable to surface modification effects, such as PEGylation or peptide conjugation. It should also be noted that DLS measurements are intensity-weighted and therefore primarily reflect larger particles, potentially underrepresenting smaller micellar populations.

### 3.6 Cytotoxicity

**Fig. 10:**
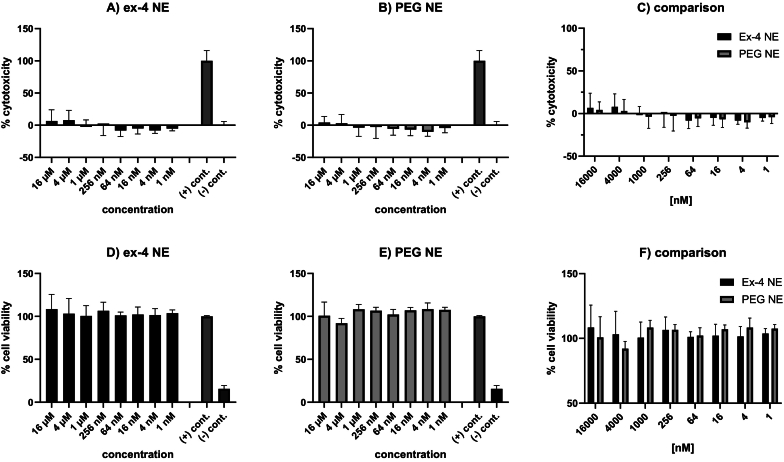
Cytotoxicity of conjugated nanoemulsions in 3T3-L1 cells. Data are mean ± SD (*n* = 6). Statistical analysis was performed using two-way ANOVA for effects of formulation and concentration, followed by Bonferroni-corrected multiple comparisons (*P* < 0.05 considered significant). No significant differences in cytotoxicity between formulations were observed.

Cytotoxicity was assessed on 3T3-L1 cells for NEs used in uptake studies (i.e., 5% cod liver oil/5% MCT batch, conjugated with either ex-4-PEG(2000)-DSPE or PEG(2000)-DSPE). As shown in Fig. 10 **(**A–B), nanoemulsions exhibited minimal toxicity toward 3T3-L1 cells, even at higher (micromolar) concentrations. No significant differences in cytotoxicity were observed between peptide-conjugated and non-peptide (control PEG) nanoemulsions at equivalent concentrations (Fig. 10C and F).

**Fig. 11:**
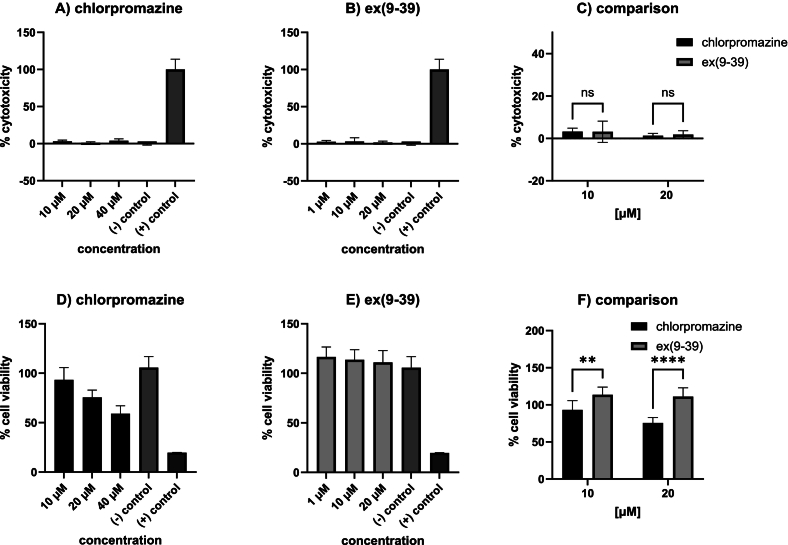
Cytotoxicity of uptake inhibitors in 3 T3-L1 cells. Data are mean ± SD (n = 6). Statistical analysis by two-way ANOVA with Bonferroni post hoc test. Significant group differences indicated as *P* < 0.01 (**) and *P* < 0.0001 (****).

The cytotoxicity profiles of inhibitors used in uptake studies were also evaluated (Fig. 11). LDH assays indicated very low or no toxicity for both exendin(9–39) and chlorpromazine at applied concentrations. However, MTS assay results revealed decreased cell metabolic activity after one hour incubation with higher chlorpromazine concentrations (Fig. 10F). This is expected, as chlorpromazine inhibits mitochondrial complex I, reducing the cellular redox activity required for MTS reduction (Hroudová and Fišar, 2011; Rana et al., 2019). LDH assay, by contrast, showed no changes in membrane integrity. Nonetheless, in uptake studies involving these inhibitors, additional nuclear staining was performed to normalize uptake signals to cell number, enabling per-cell uptake analysis.

### 3.7 Uptake over time

The uptake of exendin-4 nanoemulsion (ex-4 NE) was evaluated in 3T3-L1 cells over 24 h relative to control (PEG) NE, enabling analysis of early kinetics, area under the curve (AUC), and internalization efficiency. Uptake and saturation behavior depended on cell differentiation state, nanoemulsion concentration, and the presence of peptide (ex-4).

**Fig. 12:**
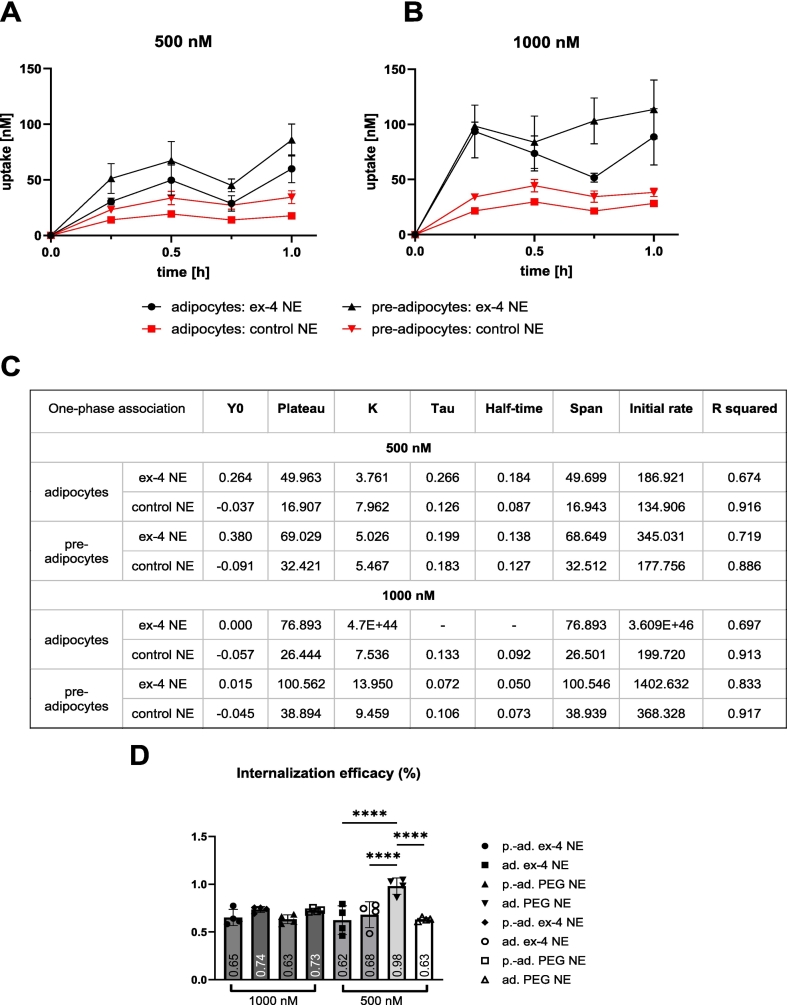
Early-phase uptake of exendin-4 nanoemulsion compared to control (PEG) nanoemulsion in two different concentrations (500 nM & 1000 nM) in 3T3-L1 pre-adipocytes and adipocytes (A − B). Data are presented as mean ± SD (*n* = 3). Calculated parameters are presented in the accompanying table (C). Internalization efficiency was determined by comparing uptake signals before and after the treatment with 0.1%TritonX/0.2 N NaOH (n = 3, each dot presents mean value per single time-point, bar represent mean value of the four time points within the first hour). Statistical analysis used one-way ANOVA with Bonferroni correction; P < 0.0001 (****) indicates significant differences.

Early-phase kinetics are shown in Fig. 12(A–C). As cellular uptake of nanocarriers generally involves sequential steps—membrane binding, endocytosis, accumulation, and saturation—a one-phase association model provides the best fit (Ruseska et al., 2025). Notably, control NEs exhibited a high model fit (R^2^ = 0.8–0.9), while exendin-4 NEs showed slightly lower fit values (R^2^ = 0.6–0.8) (Fig. 12C). Maximum uptake (Y_max_) was typically 2–3 times higher for ex-4 NE than for PEG NE, and the initial uptake rate was consistently greater for ex-4 NE across all conditions. For adipocytes exposed to 1000 nM ex-4 NE, Fig. 12C), the uptake occurred faster than could be resolved, therefore half-time values could not be reliably calculated.

Internalization efficiency, calculated as the fraction of uptake signal remaining after surface-bound nanoemulsions were removed, was approximately 70% for most conditions, indicating both internalized and surface-bound nanoparticles. Non-targeted nanoparticles at 500 nM in adipocytes showed apparent internalization efficiency near 100%, but these coincided with low total uptake and was likely unreliable due to higher variability and non-saturating dose effects. This effect was only observed in adipocytes, likely reflecting their rounded morphology and less accessible membrane area for nonspecific binding.

**Fig. 13:**
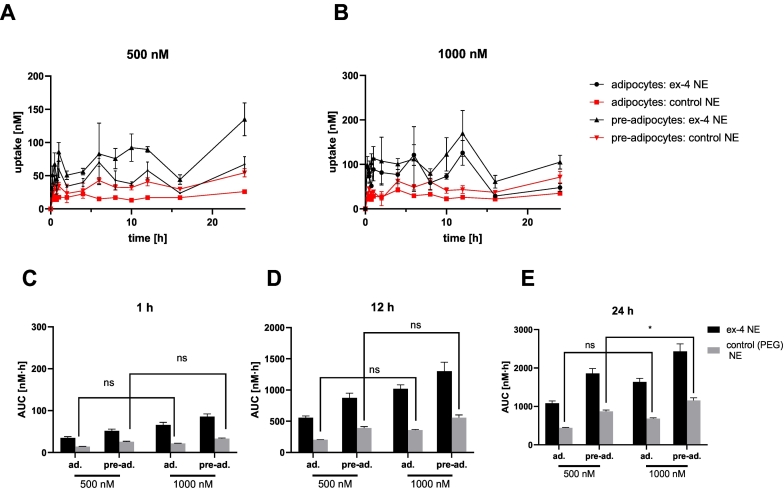
Uptake kinetics of ex-4 and PEG nanoemulsions (NE) in 3T3-L1 pre-adipocytes and adipocytes over 24 h. Uptake curves are shown in panels A–B. The corresponding area under the curve (AUC) values calculated after 1 h (C), 12 h (D), and 24 h (E) are presented. Uptake values were converted to nM equivalents using a calibration curve, and AUC values are expressed in nM·h.

Uptake curves for ex-4 and PEG NEs at both concentrations over 24 h are shown in Fig. 13(A–B). AUCs were calculated at 1, 12, and 24 h for both concentrations (Fig. 13: C–E), with statistical comparisons summarized in Table 1. Three-way ANOVA evaluated the influence of cell differentiation stage (pre-adipocyte vs. adipocyte), targeting (ex-4 NE vs. PEG NE), and concentration (500 vs. 1000 nM) on the uptake.

**Table 1:** Statistical comparison of AUCs from uptake profiles. Mean differences (nM·h) are shown with significance indicated as: P < 0.05 (), P < 0.01 (), P < 0.001 (), and P < 0.0001 (****).

		1 h		12 h		24 h	
comparison	Category	Mean Diff.	*P* summary	Mean Diff.	*P* summary	Mean Diff.	*P* summary
pre- adipocytes vs. adipocytes	ex-4 NE [500 nM]	16.86	***	318.7	***	776	****
	PEG NE [500 nM]	11.3	*	186.9	*	426	***
	ex-4 NE [1000 nM]	19.76	****	284	***	794	****
	PEG NE [1000 nM]	11.37	*	199.5	*	469.8	***
ex-4 NE vs. PEG NE	adipocytes [500 nM]	20.74	****	354	****	639.8	****
	adipocytes [1000 nM]	44.1	****	661.4	****	952.8	****
	pre-adipocytes [500 nM]	26.3	****	485.8	****	989.8	****
	pre-adipocytes [1000 nM]	52.49	****	745.9	****	1277	****
1000 nM vs. 500 nM	adipocytes: ex-4 NE	31.05	****	463.1	****	554	****
	adipocytes: PEG NE	7.69	ns	155.7	ns	241	ns
	pre-adipocytes: ex-4 NE	33.95	****	428.4	****	572	****
	pre-adipocytes: PEG NE	7.76	ns	168.3	ns	284.8	*

Ex-4 NE consistently showed higher AUC values than PEG NE across all conditions (*P* < 0.0001), with significant differences apparent already after 1 h. Uptake of ex-4 NE also differed significantly between pre-adipocytes and adipocytes (*P* < 0.001 to P < 0.0001), while PEG NE differences were less pronounced, reaching only moderate significance (*P* < 0.05 up to 12 h). Comparison of concentrations revealed minimal AUC changes for PEG NE, indicating uptake saturation. Conversely, increasing ex-4 NE concentration significantly increased AUC in all groups (P < 0.0001).

In addition to the early-phase kinetic analysis, the overall uptake profiles revealed distinct temporal patterns between ex-4 and control (PEG) nanoemulsions. PEG nanoemulsions exhibited an increase in uptake within the first hour, followed by a stable plateau, which may reflect a balance between endocytic uptake and intracellular processing under steady-state conditions. In contrast, ex-4 nanoemulsions showed a more variable, phase-like uptake pattern with successive increases over time and pronounced fluctuations, suggesting that surface functionalization with exendin-4 alters the cellular interaction and intracellular fate of the nanocarriers. This behavior may reflect a combination of factors, including altered membrane interactions, changes in endocytic trafficking dynamics, and intracellular processing such as exocytosis or lysosomal degradation.

It is also known that GLP-1 receptor undergoes ligand-induced internalization and recycling (Jones et al., 2018), and therefore it is plausible that dynamic receptor trafficking contributes to the observed time-dependent uptake patterns. Uptake maxima occurred at different time points depending on concentration, followed by a decrease at approximately 16 h. This may reflect a transient reduction in available cell surface binding sites and/or a shift in the balance between uptake and intracellular processing. In addition, biological variability and partial leakage or quenching of Nile Red fluorescence under intracellular conditions may contribute to the observed signal fluctuations. Taken together, the data indicate that ex-4 modification modulates uptake kinetics in a time-dependent manner, although the precise underlying mechanisms remain to be fully elucidated.

### 3.8 Receptor imaging

**Fig. 14:**
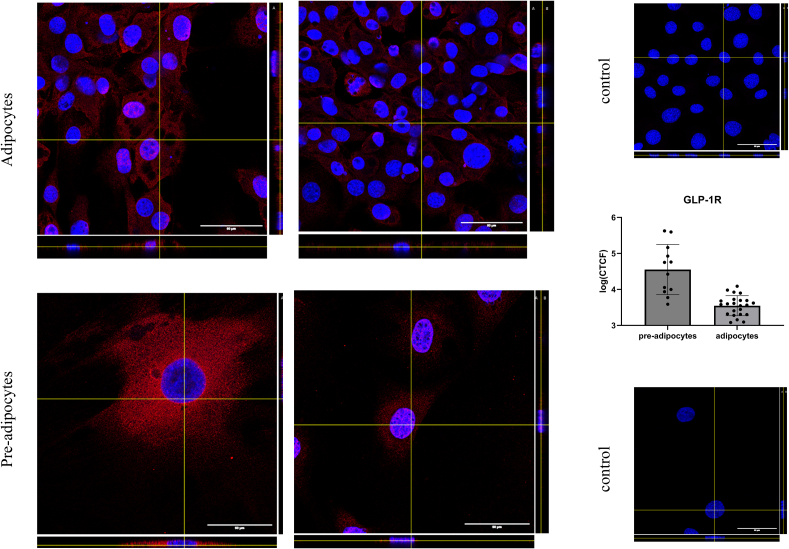
CLSM images of GLP-1R immunostaining (red) in 3T3-L1 cells before and after differentiation. Normal rabbit IgG was used as an isotype control. Images are orthogonal projections of z-stacks (B = basal, A = apical; scale bar = 50 μm). Graph shows CTCF values per cell from a single experiment. (For interpretation of the references to colour in this figure legend, the reader is referred to the web version of this article.)

GLP-1 receptor (GLP-1R) presence in 3T3-L1 cells was confirmed by immunofluorescence (Fig. 14). Staining was observed in both pre-adipocytes and adipocytes, with variable nuclear overlap between cells. The receptor was primarily cytoplasmic, consistent with previous reports in pancreas, liver, and kidney (Zhang et al., 2020). In differentiated cells, large receptor-free structures likely corresponded to lipid droplets.

Receptor staining was detected before and after 6 days of differentiation, indicating that GLP-1R is retained post-differentiation, as previously reported (Chen et al., 2017). Semi-quantitative analysis using corrected total cell fluorescence (CTCF) suggested higher receptor signal in pre-adipocytes, also consistent with literature (Chen et al., 2017); however, these results are representative and based on limited samples.

### 3.9 Uptake imaging

**Fig. 15:**
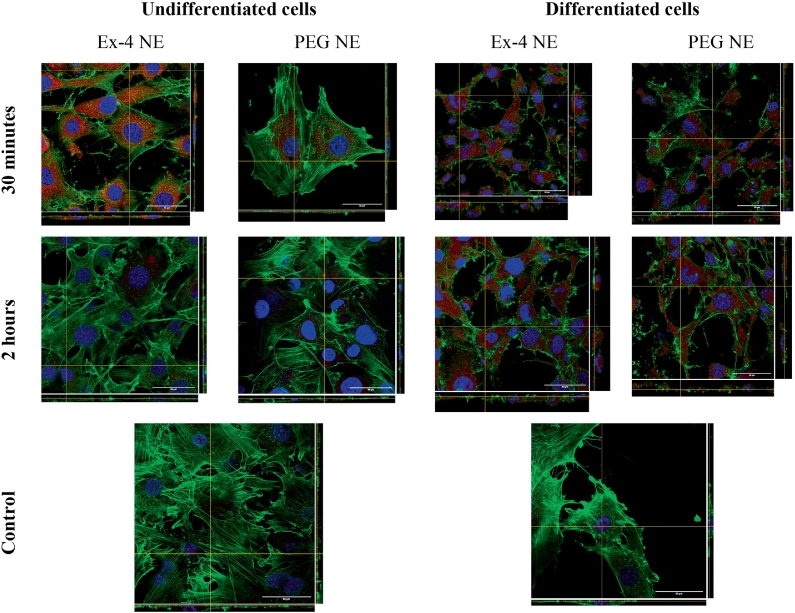
CLSM images of nanoemulsion uptake in 3T3-L1 cells before and after differentiation to mature adipocytes, together with non-incubated control cells. Images are orthogonal projections of z-stacks (B = basal side, A = apical side; scale bar = 50 μm). Nanoemulsions in red, nuclei in blue (Hoechst), F-actin in green (phalloidin AF488). (For interpretation of the references to colour in this figure legend, the reader is referred to the web version of this article.)

Confocal laser scanning microscopy (CLSM) confirmed internalization of nanoemulsion droplets in 3T3-L1 cells (Fig. 15). In pre-adipocytes, signal appeared as discrete puncta, suggesting accumulation in endocytic vesicles. In mature adipocytes, signal was more diffuse, potentially reflecting redistribution of Nile Red dye within intracellular lipid pools. F-actin organization also differed between cell types, consistent with morphological changes during differentiation. In both, nanoemulsion droplets were cytoplasmic and excluded from nuclei. Colocalization analysis assessed spatial relationships between nanoparticles and receptor.

### 3.10 Colocalization studies

**Fig. 16:**
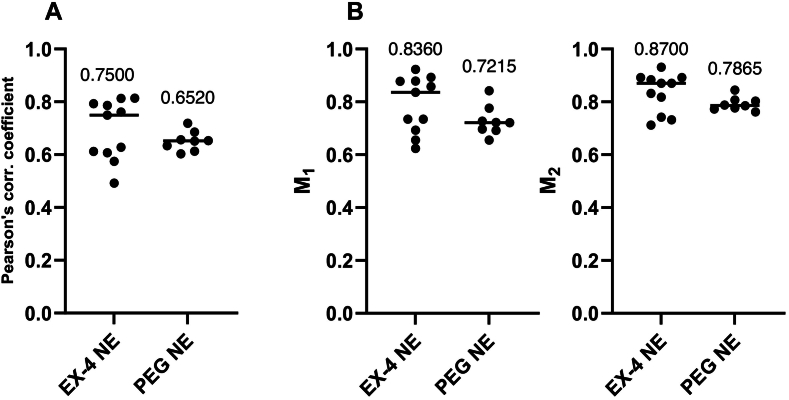
Pearson correlation coefficient (PCC) (A) and Manders' colocalization coefficients (B) calculated for nanoemulsion–receptor colocalization. Thresholding for Manders' coefficients was performed automatically using the Costes method, whereas Pearson coefficients were calculated from unthresholded intensity values. Each dot represents one analyzed z-stack image (*n* = 11 for ex-4 NE; *n* = 9 for PEG NE). Numbers indicate the median values for each group.

**Fig. 17:**
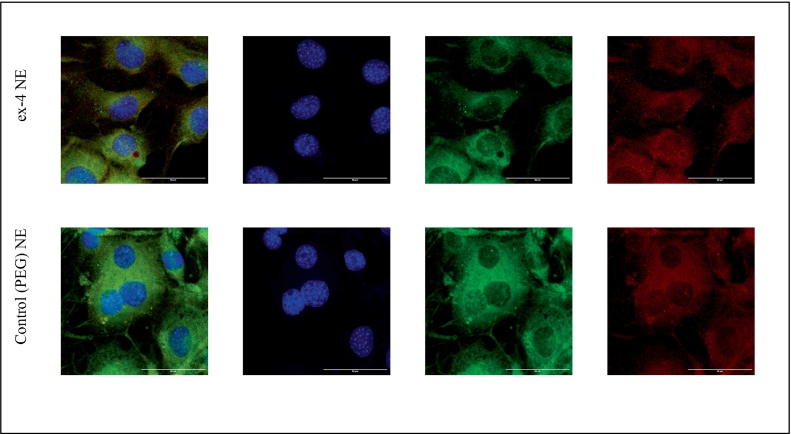
Representative CLSM images showing colocalization of GLP-1R (red) with nanoemulsion droplets (green); nuclei in blue (DAPI) (n = 1). Scale bar = 50 μm; summed intensity projections of z-stacks. (For interpretation of the references to colour in this figure legend, the reader is referred to the web version of this article.)

Colocalization analysis showed a trend toward higher colocalization of ex-4 nanoemulsions (NE) with GLP-1R compared to PEG NE, though differences were not statistically significant (Fig. 16). Manders' coefficients M_1_ and M_2_ represent NE signal overlapping with receptor signal and vice versa, calculated with Costes thresholding. Both nanoemulsions showed relatively high colocalization (median PCC: 0.75 vs. 0.65; M1: 0.84 vs. 0.72; M2: 0.87 vs. 0.79 for ex-4 NE vs. PEG NE). Representative images (Fig. 17) showed heterogeneous intracellular distribution and both overlapping and exclusive signal in vesicles, suggesting alternative uptake pathways. Cell variability and rapid receptor internalization/desensitization may also influence colocalization during the incubation period.

### 3.11 Uptake with inhibitors

**Fig. 18:**
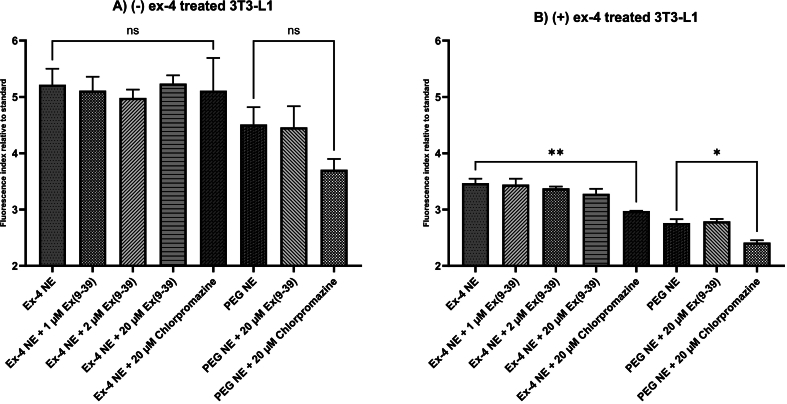
Nanoemulsion uptake in the presence of inhibitors. Uptake normalized to DAPI and BODIPY standard signals and divided by 1000, reflecting per-cell normalized uptake. Uptake values were calculated as the ratio of NE fluorescence signal to the product of DAPI signal and the BODIPY standard signal (500 nM NE) divided by 1000, thereby reflecting nanoparticle uptake normalized to both the standard and cell number. One-way ANOVA (*n* = 3); *P* < 0.05 (*); *P* < 0.01 (**). Inhibitor concentrations represent the final concentrations after addition of the NE (end concentration 1000 nM).

To elucidate mechanisms of NE uptake, GLP-1R antagonist exendin(9–39) and chlorpromazine (clathrin-mediated endocytosis inhibitor) were used. GLP-1R internalization involves both clathrin- and caveolae-mediated pathways (Von Moo et al., 2025). Results (Fig. 18) showed reduced NE uptake in adipocytes treated with exendin-4 during differentiation, while exendin(9–39) did not significantly alter uptake of either NE type. Chlorpromazine significantly reduced uptake in exendin-4–treated adipocytes for both nanoemulsions; in untreated adipocytes, only PEG NE uptake was modestly reduced by chlorpromazine, though without statistical significance.

### 3.12 Effects on lipid accumulation

Lipid accumulation was analyzed after 10 days of differentiation. Firstly, the effects of free exendin-4 on adipogenesis were evaluated by treating the cells from day 0 of differentiation protocol. In a second experimental setup, free ex-4, ex-4 NE, and control PEG NE were added from day 2 of differentiation. In both cases, the tested substances were subsequently reapplied every second day. Results were compared to untreated controls.

**Fig. 19:**
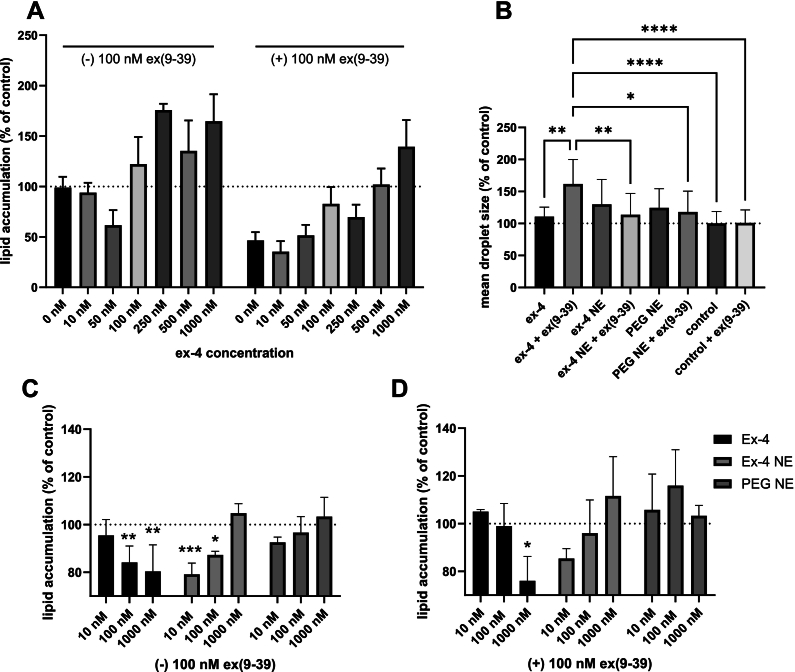
Oil Red O (ORO) staining analysis of lipid accumulation in 3T3-L1 cells treated either at pre-adipocyte stage (A) or from day 2 of differentiation (B—D). Results expressed as % ratio to untreated controls (100% = dashed line). Statistical analysis: one-way ANOVA (B), two-way ANOVA with Bonferroni correction (C—D); n = 3; P < 0.05 (*), P < 0.01 (**), *P* < 0.001 (***), *P* < 0.0001 (****). (For interpretation of the references to colour in this figure legend, the reader is referred to the web version of this article.)

As previously reported, ex-4 treatment from induction phase (day 0) increased lipid accumulation (Chen et al., 2017). When applied from day 2, lipid accumulation decreased in a concentration-dependent manner. These effects were partially reversed by exendin(9–39), indicating receptor-mediated mechanisms (Fig. 19). PEG NE showed concentration-dependent effects; at low concentration, lipid accumulation was reduced, at intermediate it was comparable to control, and at high concentration moderately increased. Ex-4 NE strongly reduced lipid accumulation at low concentrations, surpassing the anti-lipogenic effect of high concentrations of free ex-4; at higher NE concentrations, this effect diminished and lipid accumulation returned toward control levels. In presence of exendin(9–39), lipid accumulation increased for both ex-4 and PEG NEs, implying broader metabolic shifts under receptor inhibition.

Lipid droplet size was assessed under selected conditions (100 nM test substance and/or 100 nM exendin(9–39)) (Fig. 19B). Significant increase in droplet size was seen only with combined free ex-4 and exendin(9–39); NE-treated groups did not show significant changes, with droplet size comparable to control.

## 4 Discussion

In this study, we developed an exendin-4-functionalized nanoemulsion prepared by high-pressure homogenization and evaluated its physicochemical properties, cellular uptake, and biological activity in 3T3-L1 cells. From a physiological perspective, lipid-based nanocarriers are often conceptually compared to endogenous lipoproteins such as chylomicrons, which are formed in the intestine following dietary lipid intake and transported via the lymphatic system. Chylomicrons typically range from approximately 75 to 1200 nm in diameter and serve as natural carriers for hydrophobic compounds. This analogy is frequently used to rationalize the design of lipid-based delivery systems. However, intravenously administered nanoemulsions do not follow the same biological pathways as enterally formed chylomicrons, and their in vivo behavior is primarily governed by interactions with plasma proteins and uptake by the mononuclear phagocyte system (MPS) (Trinick and Duly, 2012). In this context, particle size plays a critical role in determining both biodistribution and safety. Larger droplets, particularly in the micrometer range, are associated not only with an increased risk of embolism but also with enhanced uptake by the MPS, leading to accumulation in organs such as the liver and spleen. This may affect metabolic clearance, particularly via hepatic pathways, and has been associated with adverse effects including oxidative stress and potential impairment of immune cell function (Driscoll, 2017). Conversely, very small particles may undergo rapid clearance, limiting circulation time and reducing targeting efficiency (Hosseini-Kharat et al., 2025). Therefore, maintaining a controlled particle size distribution is essential not only from a safety perspective but also for achieving the desired pharmacokinetic profile. For all tested formulations, the *Z*-average size ranged from 150 to 200 nm, with PdI values between 0.10 and 0.20. Moreover, the zeta potential of the raw NEs was between −40 mV and − 50 mV, consistent with good colloidal stability.

Cod liver oil, MCTs, and egg lecithin (E80 SN) were selected for preparation of NEs. E80 SN contains a relatively high proportion of phosphatidylethanolamine (PE) compared to other emulsifier mixtures. Due to its smaller headgroup and cone-shaped geometry, PE promotes tighter packing at the oil–water interface, increasing local membrane order while maintaining interfacial fluidity. Its intrinsic negative curvature may also facilitate fusion-related processes. In contrast, phosphatidylcholine (PC), with its larger and more hydrated cylindrical headgroup, stabilizes planar interfaces and enhances resistance to droplet coalescence (Martindale et al., 2025; van der Veen et al., 2017). The PC:PE ratio of E80 SN (∼70% PC / ∼30% PE) supports nanoemulsion stability while broadly resembling physiological membrane compositions (Lee et al., 2018). While cellular PC:PE remodeling is primarily regulated by intracellular metabolism rather than exogenous lipid supply, its association with metabolic states such as insulin sensitivity highlights the biological relevance of PE-rich environments (Lee et al., 2018). In the present study, however, the elevated PE content was selected primarily for its favorable interfacial properties.

High-pressure homogenization followed by pH adjustment (8.5) proved to be a robust method for preparing stable nanoemulsions across different oil compositions. Among the tested formulations, cod liver oil provided optimal stability. In addition, marine oils are a source of ω-3 fatty acids, which are widely reported to exert anti-inflammatory and metabolic effects, including modulation of lipid metabolism in adipocytes (Das et al., 2026; Hernandez et al., 2021; Ide, 2023; Phadtare et al., 2021).

A lipid phase consisting of 50% cod liver oil and 50% medium-chain triglycerides (MCTs) was selected for further studies. MCTs are commonly combined with long-chain triglycerides (LCTs) in parenteral emulsions (e.g., Lipuro® formulations) to improve physical stability, reduce injection-related adverse effects, and enhance metabolic clearance, as MCTs are more rapidly hydrolyzed and eliminated from circulation (Bach et al., 1989; Yamakage et al., 2005). Pharmacopoeial-grade MCTs typically contain caprylic acid (C8) and capric acid (C10), which have been reported to act as partial agonists of PPARγ. These effects are, however, concentration-dependent and are more evident at lower concentrations, whereas higher concentrations likely influence multiple metabolic pathways beyond PPARγ signaling (Liberato et al., 2012).

The coupling efficiency of exendin-4 to DSPE-PEG(2000) via maleimide–thiol reaction reached approximately 97%. Also, PEGylated and ex-4-functionalized NEs were successfully prepared, showing the expected slight increase in hydrodynamic diameter (5–10 nm) following surface modification. The selected PEG and ligand surface densities were based on literature reports (Hak et al., 2012) and our preliminary studies on comparable nanoemulsion formulations, aiming to balance colloidal stability with ligand accessibility while minimizing steric hindrance at the droplet surface. PEGylated NEs exhibited a slightly more negative zeta potential, whereas peptide-conjugated nanoemulsions showed a shift toward less negative values, likely due to steric shielding and the presence of residual HEPES buffer, which increases ionic strength and can compress the electrical double layer. Over time, the zeta potential of ex-4 NE slightly decreased, indicating reduced long-term stability; therefore, freshly prepared formulations were used for in vitro experiments.

Cryo-TEM analysis revealed no major structural differences between raw and conjugated nanoemulsions. Both systems consisted primarily of oil droplets, accompanied by a minor population of vesicular structures consistent with liposomes or micelles. However, micelle formation did not appear to be the dominant structural change following conjugation.

Fluorescence imaging confirmed cellular uptake and cytoplasmic localization of both formulations. In agreement with previous reports (Chen et al., 2017), GLP-1 receptor expression decreased during adipocyte differentiation. Colocalization analysis indicated only a trend toward increased overlap between exendin-4 NE and GLP-1R compared to the PEGylated control, without reaching statistical significance. This may be attributed to cellular heterogeneity in receptor expression, the contribution of alternative uptake pathways, and the potential partial dissociation of the ligand from the nanoemulsion interface. Moreover, ligand dissociation kinetics within endosomes may influence GLP-1R trafficking: lower dissociation rates have been associated with prolonged receptor retention in endosomal compartments, favoring lysosomal degradation over receptor recycling (Marzook et al., 2021). In addition, although antibodies against GLP-1R should not be considered orthosteric ligands, treatment with ex-4 NE may induce steric hindrance that affects receptor staining.

Quantitative uptake studies demonstrated that exendin-4 nanoemulsions exhibited higher initial uptake rates and area under the curve (AUC) values compared to PEGylated controls. Uptake was also influenced by differentiation status, with higher uptake observed in pre-adipocytes. While exendin-4-NE showed concentration-dependent uptake, PEGylated NE did not, suggesting saturation of non-specific uptake pathways. Moreover, Ex-4 nanoemulsions exhibited more dynamic uptake patterns, which may reflect altered cellular interactions and intracellular trafficking dynamics associated with surface functionalization, potentially involving receptor-mediated processes.

Mechanistic studies using exendin(9–39) and chlorpromazine indicated that nanoemulsion uptake is not exclusively mediated by GLP-1R but is strongly influenced by the cellular signaling state. Chronic exposure to exendin-4 during adipogenesis shifted uptake toward clathrin-mediated pathways, as evidenced by increased sensitivity to chlorpromazine. This suggests that sustained GLP-1R activation modulates the balance between clathrin-dependent and clathrin-independent endocytosis. In mature adipocytes, which are enriched in caveolae structures stabilized by caveolin-1, clathrin-independent pathways typically dominate. However, under conditions of altered signaling or lipid availability, endocytic routing can shift (Briand et al., 2014; Hinze and Boucrot, 2018). Similar behavior has been described for GLUT4 trafficking, where insulin stimulation promotes a transition toward clathrin-mediated internalization (Hinze and Boucrot, 2018). Additionally, CD36-mediated fatty acid uptake has been shown to switch from caveolae-associated pathways at low fatty acid concentrations to clathrin-mediated uptake under high lipid conditions (Singh et al., 2025). These findings support the concept that nanoemulsion uptake is governed by dynamic regulation of endocytic pathways rather than a single dominant mechanism.

The effects of nanoemulsion uptake were further evaluated by assessing lipid accumulation in differentiating adipocytes. When free exendin-4 was applied from the onset of differentiation, lipid accumulation increased, consistent with its role in promoting early adipogenic commitment. In contrast, when applied from day 2 onward, exendin-4 reduced lipid accumulation, reflecting stage-dependent effects of GLP-1R signaling (Chen et al., 2017; Yang et al., 2013). Exendin-4 NEs exhibited a markedly enhanced anti-lipogenic effect compared to free ligand, with 10 nM nanoemulsion-bound exendin-4 producing effects comparable to 1000 nM of free exendin-4. This likely reflects increased local ligand density and prolonged interaction with the cell surface (Briand et al., 2014; Hinze and Boucrot, 2018).

Importantly, the control NE also reduced lipid accumulation at low concentrations, albeit less pronounced than that observed for ex-4 NE at equivalent concentrations. At higher concentrations, an increased lipid storage was observed for both NEs, consistent with substrate-driven effects. Similar concentration-dependent effects of fatty acids and marine oils on adipogenesis have been reported (Das et al., 2026; Lim and Chien, 2024; Phadtare et al., 2021). Inhibition of GLP-1R signaling increased lipid accumulation across all conditions, suggesting that basal receptor activity contributes to the regulation of lipid storage.

Overall, these findings demonstrate that nanoemulsions modulate lipid metabolism in adipocytes in a concentration-dependent manner, and that GLP-1R activation can counteract lipid accumulation at lower lipid loads but is overridden at higher nanoemulsion concentrations. Functionalization with exendin-4 enhanced cellular uptake and amplified biological activity, while the lipid matrix itself contributed additional metabolic effects. Importantly, the observed biological responses indicate that the surface-bound exendin-4 retained functional activity after conjugation and incorporation into the nanoemulsion formulation. However, the present study does not provide direct evidence of receptor binding affinity or unequivocal proof of receptor-mediated uptake. Future studies should therefore focus on quantitative receptor-binding analyses and on elucidating the relative contributions of GLP-1R-dependent and alternative uptake pathways in different cell types with varying GLP-1R expression levels. In addition, systematic evaluation of ligand surface density will be important to determine the optimal concentration for uptake efficiency.

Beyond ligand delivery, the nanoemulsion platform offers the possibility to incorporate hydrophobic therapeutics, enabling combination treatment strategies. Furthermore, future applications may extend beyond parenteral administration to subcutaneous and oral delivery systems. The DSPE-PEG anchor used for exendin-4 conjugation resembles the fatty acid modification employed in liraglutide and semaglutide, where lipidation markedly improves pharmacokinetics through albumin binding and prolonged circulation. In this context, nanoemulsions may provide a lipid-based carrier for peptide delivery, with potential to enhance gastrointestinal stability and absorption when combined with protective polymer coatings, thereby reducing reliance on permeation enhancers.

## 5 Conclusion

Overall, our data indicate that the uptake of exendin-4 nanoemulsion (ex-4 NE) in 3T3-L1 cells is not primarily driven by classical GLP-1R–mediated internalization. Inhibition studies and colocalization analysis showed only minimal receptor-dependent uptake, suggesting that alternative pathways—such as caveolae-mediated endocytosis or macropinocytosis—dominate in adipocytes.

Despite this, ex-4 NE consistently exhibited higher cellular uptake than non-targeted PEG nanoemulsions, indicating that surface-bound exendin-4 can modulate cellular lipid handling and indirectly enhance nanoparticle internalization. This effect was observed in both preadipocytes and mature adipocytes, supporting a metabolism-dependent, ligand-driven enhancement of uptake rather than strict receptor specificity. Importantly, nanoemulsion-mediated delivery significantly increased the functional potency of exendin-4 compared to the free peptide, leading to stronger effects on lipid accumulation.

Future studies should investigate the receptor-binding properties and affinity of the DSPE-PEG-exendin-4 construct in greater detail to better understand the contribution of GLP-1R interactions to the observed biological effects. While adipocytes exhibit low GLP-1 receptor expression, other metabolically active tissues such as pancreatic β-cells may provide more direct receptor-mediated targeting opportunities (Wang et al., 2014). In parallel, the intrinsic metabolic activity of nanoemulsion lipid components—including ω-3 fatty acids and medium-chain triglycerides—may offer an additional modulatory layer, suggesting potential for future combination strategies targeting lipid metabolism in obesity-related disorders. This supports the potential of ligand-functionalized nanoemulsions as a platform for GLP-1–based therapeutic delivery strategies, with additional metabolic modulation arising from the lipid components, which should be further explored in the context of obesity and metabolic diseases.

## CRediT authorship contribution statement

**Natasa Holler:** Writing – original draft, Methodology, Investigation, Conceptualization. **Bernd Gesslbauer:** Methodology, Investigation, Formal analysis. **Carmen Tam-Amersdorfer:** Methodology, Investigation. **Ilse Letofsky-Papst:** Methodology, Investigation. **Claudia Mayrhofer:** Methodology, Investigation. **Fritz Andreae:** Methodology, Investigation. **Andreas Zimmer:** Writing – review & editing, Supervision, Funding acquisition.

## Declaration of competing interest

The authors declare the following financial interests/personal relationships which may be considered as potential competing interests:

Andreas Zimmer reports financial support was provided by Phospholipid Research Center. If there are other authors, they declare that they have no known competing financial interests or personal relationships that could have appeared to influence the work reported in this paper.

## Data Availability

All data generated or analyzed during this study are included in this article and its supplementary information. Additional raw data are available from the corresponding author upon request.
