# Supplementary material for: Peptide-functionalized nanoemulsions with exendin-4 as a model ligand
Source: Int J Pharm X. 2026 Jul 2;12:100598. doi: 10.1016/j.ijpx.2026.100598 (PMC13380532; doi:10.1016/j.ijpx.2026.100598)
Supplement: Supplementary file 1 [file mmc1.docx]

Supplementary data

1. Materials
   1. Cell culture: proliferation and differentiation of 3T3-L1 fibroblasts:

For cell cultivation and differentiation, the following substances were used:

- low glucose Dulbecco's Modified Eagle Medium (lgDMEM 1g/L, REF 11880-028, Thermo Fischer Scientific);
- high glucose Dulbecco's Modified Eagle Medium (hgDMEM 4.5g/L D-glucose, REF 41965-039, Thermo Fischer Scientific);
- fetal bovine serum (FBS, REF F7524, Sigma Aldrich);
- HEPES 1M (REF 15630-056, Thermo Fischer Scientific);
- L-glutamine 200mM (REF 25030-081, Thermo Fischer Scientific);
- Penicillin-Streptomycin (5,000 U/mL) (REF 15140-122, Thermo Fischer Scientific);
- Dexamethasone (REF D4902, Sigma Aldrich);
- IBMX (REF I7018 , Sigma Aldrich);
- Insulin human 10mg/mL (REF I9278, Sigma Aldrich).
  1. ORO staining
- 37% formalin for histology (Sigma Aldrich REF 1.03999);
- ORO stain (Sigma Aldrich, REF O0625);
- Isopropanol HPLC-grade (Car Roth, REF 9866.6);
- MilliQ water (Milli-Q® Gradient, Millipore).
  1. Uptake studies
- BODIPY 493/503 (Invitrogen, ThermoFisher Sci. REF D3922);
- DAPI (Invitrogen, ThermoFisher Sci. REF D3571);
- Hoechst 33342 (Invitrogen, ThermoFisher Sci. REF H21492);
- NileRed (Sigma Aldrich, REF 19123);
- AF488 phalloidin (Invitrogen, ThermoFisher Sci. A12379);
- TritonX (Sigma Aldrich, REF T8787);
- Chlorpromazine HCl (Sigma Aldrich, REF C8138);
- 4% Formaldehyde (Sigma Aldrich, REF HT50-1-2);
  1. Coupling reaction
- TCEP (Sigma Aldrich, REF C4706);
- NaOH (Carl Roth, REF 6771.1);
- HEPES (Sigma Aldrich, REF H3375);
- DMSO (Sigma Aldrich, REF D2438);
- L-cysteine (Sigma Aldrich, REF 30089).
  1. Immunostaining
- 1° antibody: rabbit anti-Glucagon-like peptide-1 receptor (Novus Biologicals, REF NLS1205);
- 2° antibody: Alexa Fluor 594 donkey anti-rabbit IgG Clone Poly4064 (BioLegend, REF 406418);
- Negative control: normal Rabbit IgG [stock conc. 0.1mg/mL] (Calbiochem®, Merck Millipore, REF NI01)
- Antibody diluent (Dako, Agilent, REF S3022);
- Mounting Medium (Dako, Agilent, REF S3023);
- Paraformaldehyde (Sigma Aldrich, REF 158127);
- 8-Chamber Flexiperm (Sarstedt, REF 94.6032.039).

1. Formulation

Formulation of the selected raw NE (% w/w):

5% cod liver oil (=LCT);

5% MCT;

2.4% glycerol;

1% E80 SN;

ad 100% MQ water.

1. Stability studies
   1. Overview of stability of **raw** NEs 7 and 21 days after preparation
      1. Formulations with different emulsifiers (oil phase: soybean oil/ LCT:MCT=1:1)


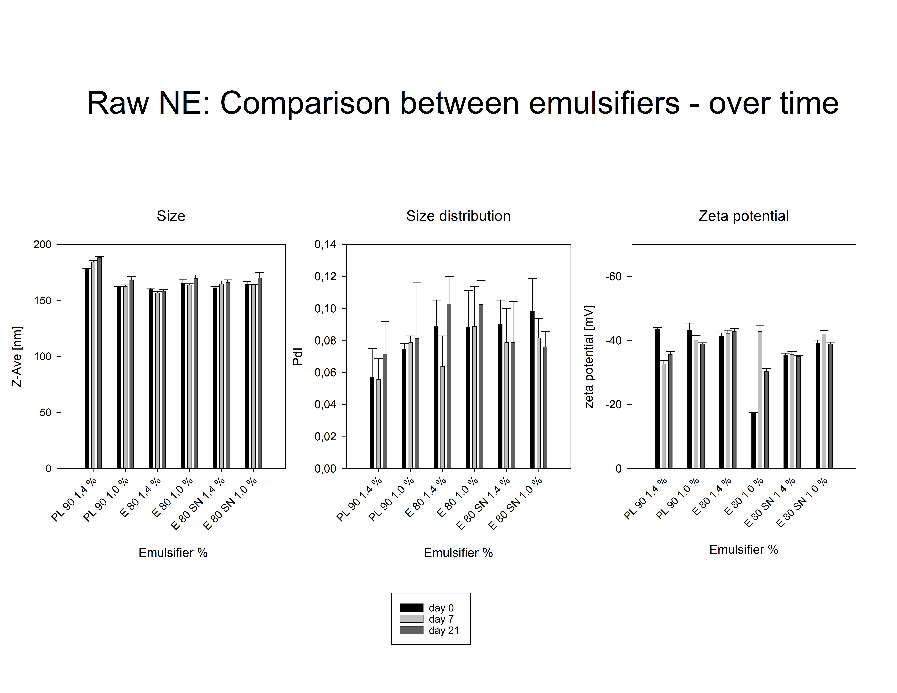


Figure S1: Comparison of mean oil droplet size, size distribution and charge for the NEs prepared with three different emuslifiers in two different concentrations. The NEs were measured on the day of preparation, and again 7 and 21 days after preparation. (mean values±SD, n=3)

- - 1. Formulations with cod liver oil


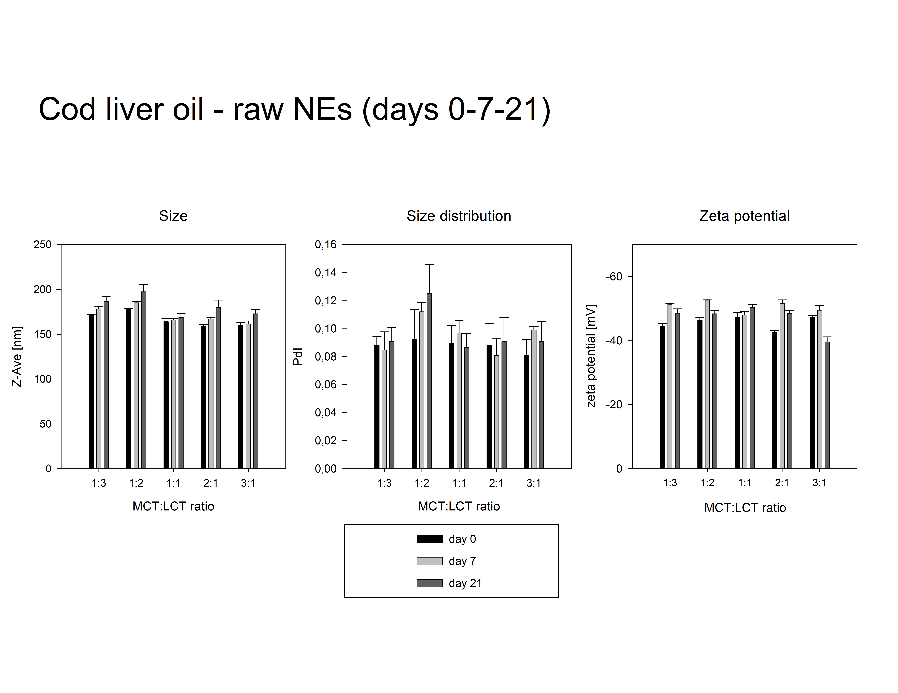


Figure S2: Comparison of mean oil droplet size, size distribution and charge for the NEs prepared with cod liver oil in 5 different MCT:LCT mass ratios. The NEs were measured on the day of preparation, and again 7 and 21 days after preparation. (mean values±SD, n=3)

- - 1. Formulations with soybean oil


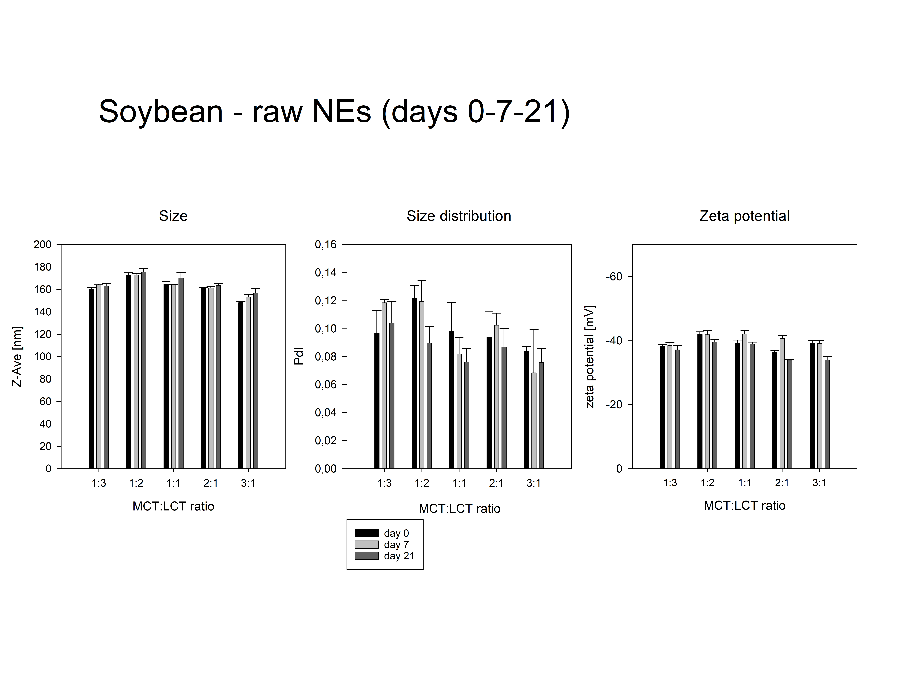


Figure S3: Comparison of mean oil droplet size, size distribution and charge for the NEs prepared with soybean oil in 5 different MCT:LCT mass ratios. The NEs were measured on the day of preparation, and again 7 and 21 days after preparation. Represented are mean values ± SD (n=3).

- - 1. Formulations with olive oil


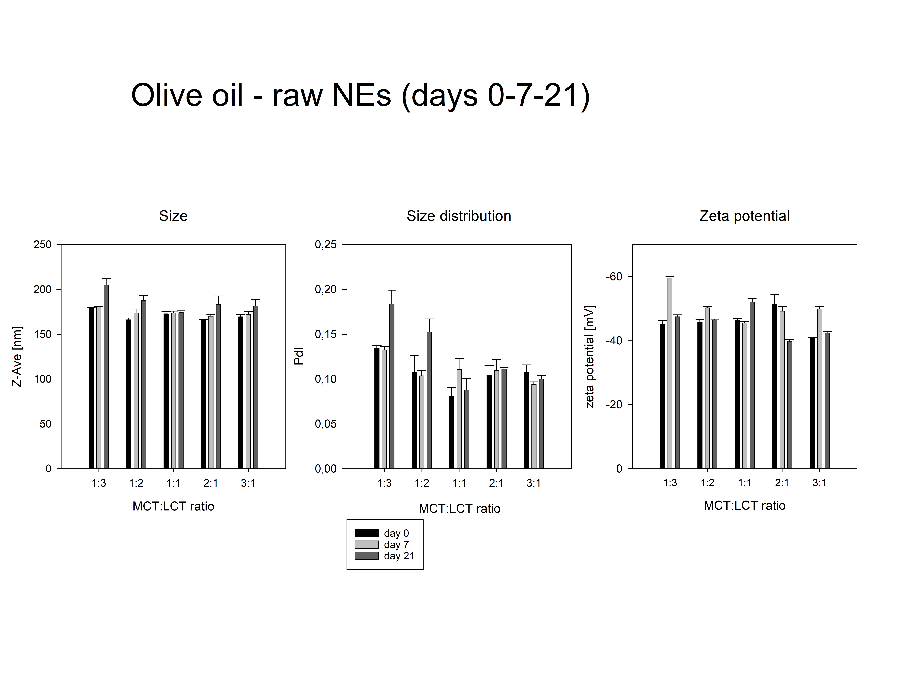


Figure S4: Comparison of mean oil droplet size, size distribution and charge for the NEs prepared with olive oil in 5 different MCT:LCT mass ratios. The NEs were measured on the day of preparation, and again 7 and 21 days after preparation. Represented are mean values ± SD (n=3).

- - 1. Formulations with castor oil


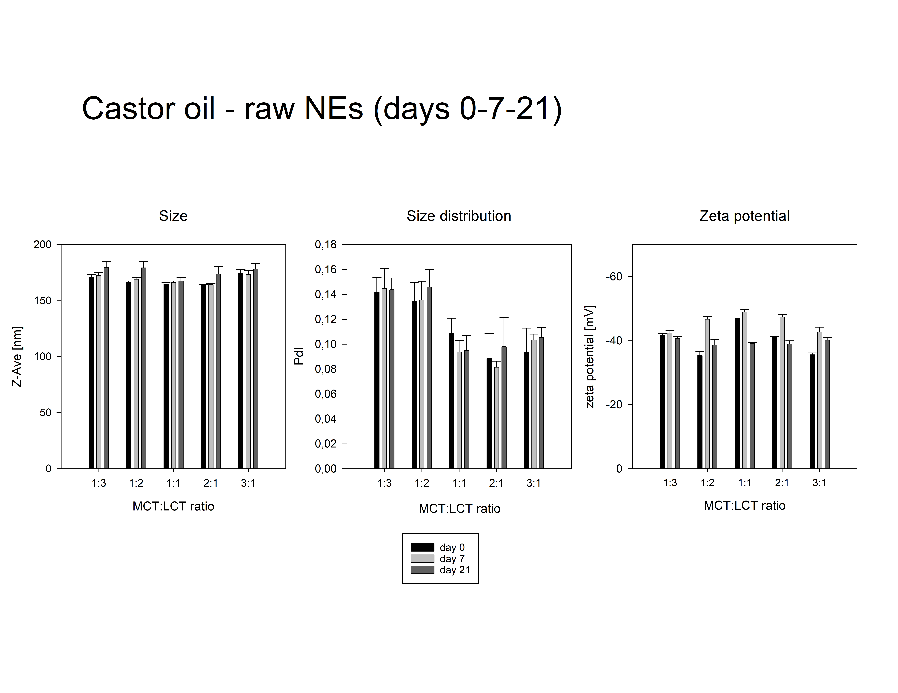


Figure S5: Comparison of mean oil droplet size, size distribution and charge for the NEs prepared with castor oil in 5 different MCT:LCT mass ratios. The NEs were measured on the day of preparation, and again 7 and 21 days after preparation. Represented are mean values ± SD (n=3).

- 1. Overview of stability of **pegylated** (PEG) NEs on the day of conjugation (day 0) and after 14 days
     1. Formulations with different emulsifiers (oil phase: soybean oil/ LCT:MCT=1:1)


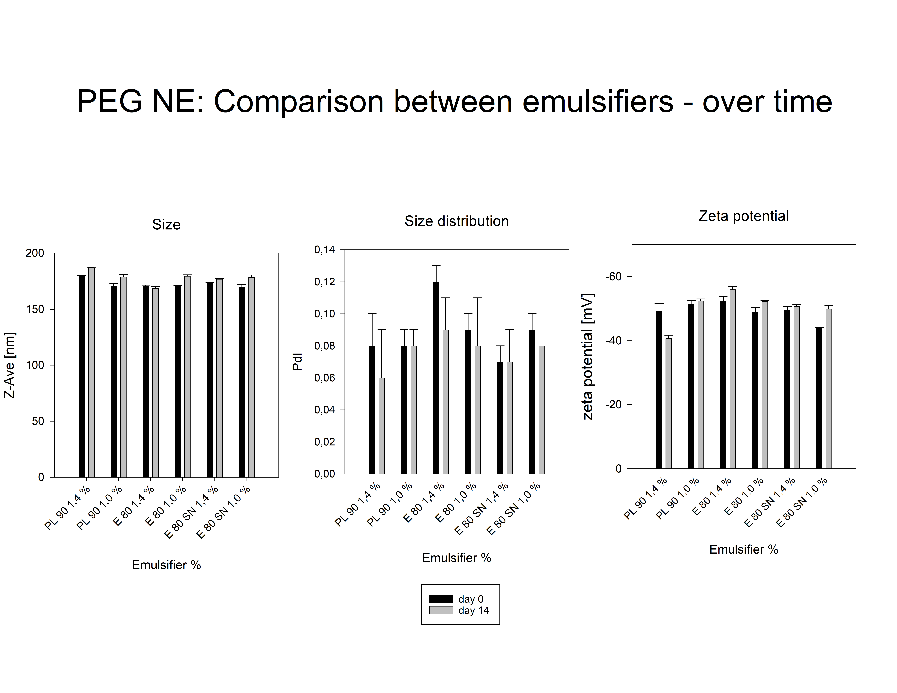


Figure S6: Comparison of mean oil droplet size, size distribution and charge for the PEG NEs prepared with three different emuslifiers in two different concentrations. The PEG NEs were measured on the day of conjugation and after 14 days. (mean values±SD, n=3)

- - 1. Formulations with cod liver oil
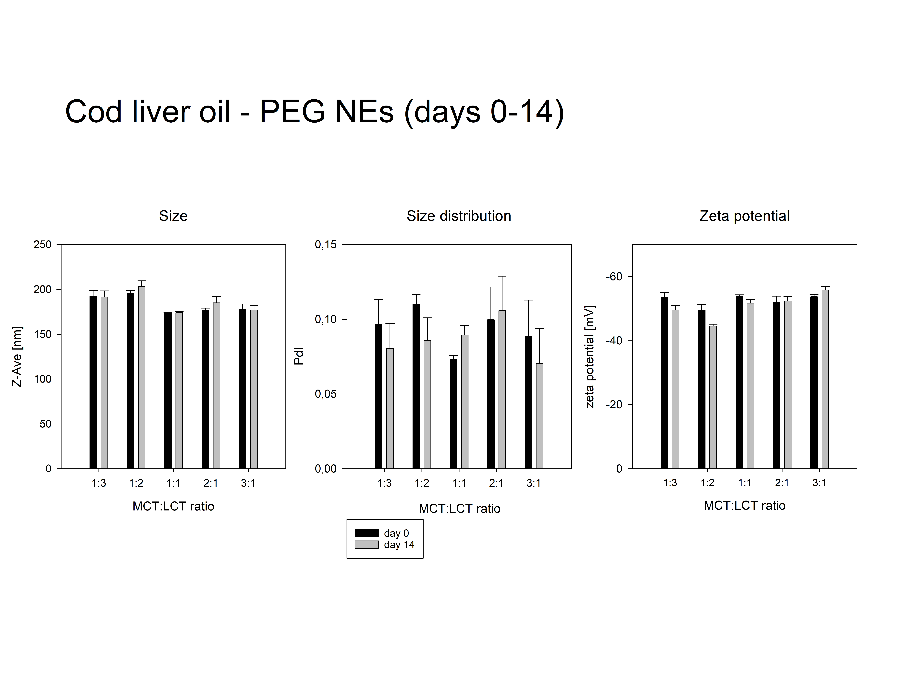


Figure S7: Comparison of mean oil droplet size, size distribution and charge for the PEG NEs prepared with cod liver oil in 5 different MCT:LCT mass ratios. The PEG NEs were measured on the day of conjugation and after 14 days. Represented are mean values ± SD (n=3).

- - 1. Formulations with soybean oil
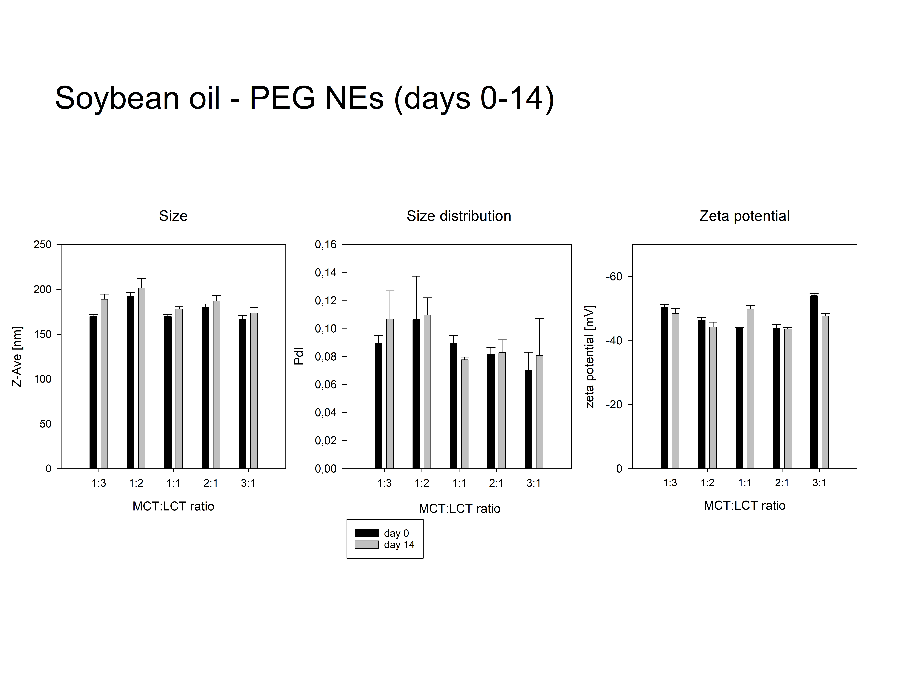


Figure S8: Comparison of mean oil droplet size, size distribution and charge for the PEG NEs prepared with soybean oil in 5 different MCT:LCT mass ratios. The PEG NEs were measured on the day of conjugation and after 14 days. Represented are mean values ± SD (n=3).

- - 1. Formulations with olive oil
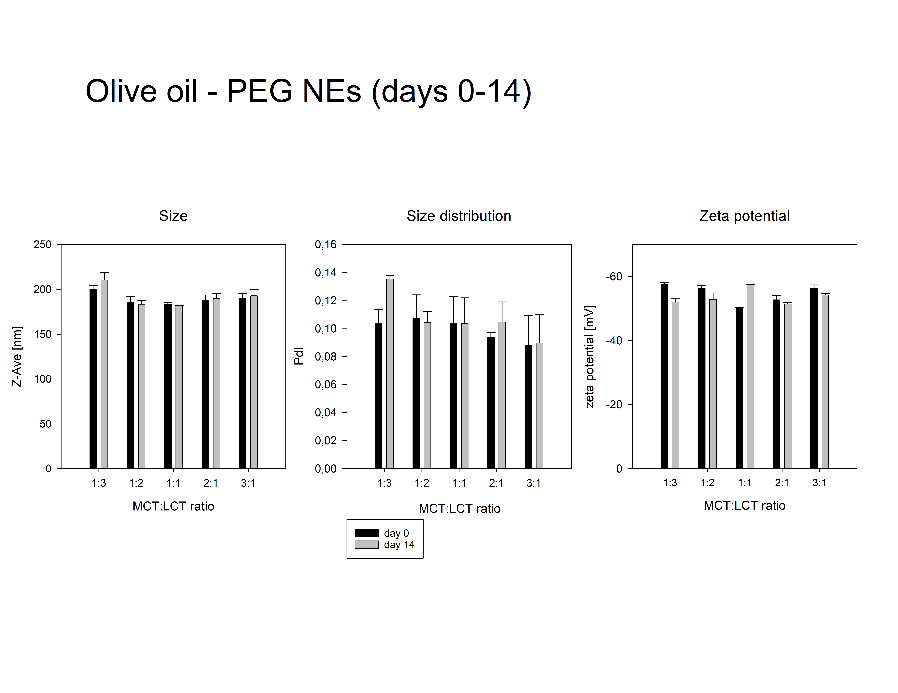


Figure S9: Comparison of mean oil droplet size, size distribution and charge for the PEG NEs prepared with olive oil in 5 different MCT:LCT mass ratios. The PEG NEs were measured on the day of conjugation and after 14 days. Represented are mean values ± SD (n=3).

- - 1. Formulations with castor oil
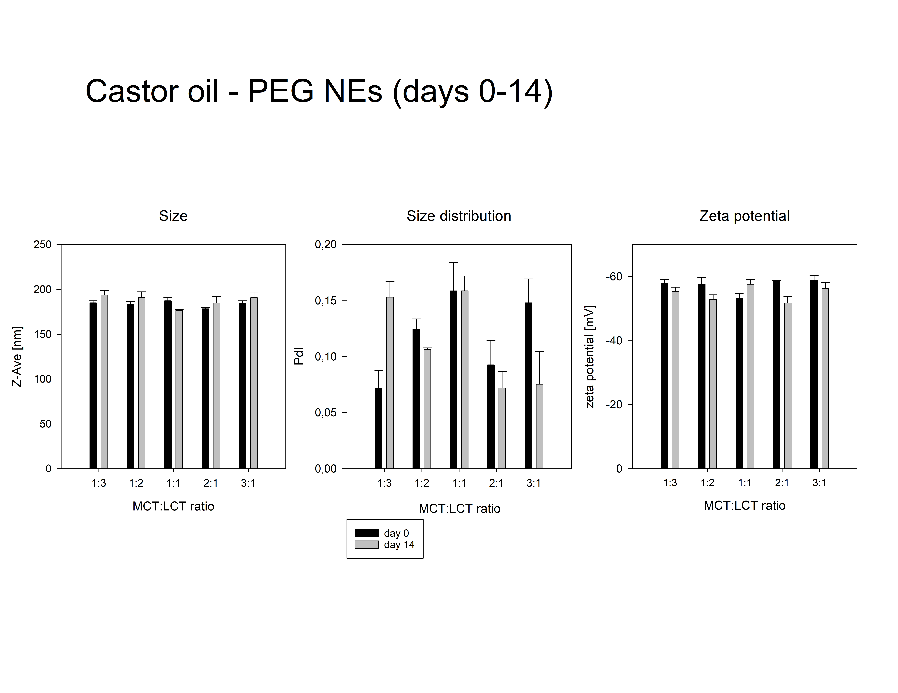


Figure S10: Comparison of mean oil droplet size, size distribution and charge for the PEG NEs prepared with castor oil in 5 different MCT:LCT mass ratios. The PEG NEs were measured on the day of conjugation and after 14 days. Represented are mean values ± SD (n=3).
